# Bioinspired and Biodegradable Hydrothermally Treated Cellulose Nanocrystal Aerogels for High Efficiency Solar Steam Generation and Sustainable Water Purification

**DOI:** 10.1002/smll.202508897

**Published:** 2025-10-30

**Authors:** Zongzhe Li, Stephanie Co, James G. Drummond, D. Mark Martinez, Feng Jiang, Mark J. MacLachlan

**Affiliations:** ^1^ Department of Chemistry University of British Columbia 2036 Main Mall Vancouver BC V6T 1Z1 Canada; ^2^ Department of Chemical and Biological Engineering University of British Columbia 2360 East Mall Vancouver BC V6T 1Z3 Canada; ^3^ Pulp and Paper Centre University of British Columbia 2385 East Mall Vancouver BC V6T 1Z4 Canada; ^4^ Sustainable Functional Biomaterials Laboratory Department of Wood Science University of British Columbia 2424 Main Mall Vancouver BC V6T 1Z4 Canada; ^5^ Stewart Blusson Quantum Matter Institute University of British Columbia 2355 East Mall Vancouver BC V6T 1Z4 Canada; ^6^ WPI Nano Life Science Institute Kanazawa University Kanazawa 920–1192 Japan; ^7^ Bioproducts Institute University of British Columbia 2360 East Mall Vancouver BC V6T 1Z3 Canada

**Keywords:** aerogels, cellulose nanocrystals, hydrothermal treatment, solar steam generation, water purification

## Abstract

Access to clean water is crucial for human survival, yet a significant portion of the global population continues to face challenges from water scarcity and contamination. Traditional water purification methods such as desalination and distillation are energy‐intensive, necessitating the adoption of green alternatives to reduce greenhouse gas emissions and mitigate global warming. High‐efficiency solar steam generation has emerged as a promising solution. Although many solar evaporators have shown impressive evaporation rates, construction of these using only sustainable materials remains challenging. Inspired by the microstructure of natural wood, a series of hydrothermally‐treated cellulose nanocrystal aerogels (HTCAs) is proposed as efficient and eco‐friendly solar steam generators. The HTCAs are prepared through a one‐pot hydrothermal treatment, avoiding the use of hazardous chemicals, and entirely based on sustainable cellulose nanocrystals. They present low tortuosity porous microstructures and exhibit high evaporation rate (1.70 kg m^−2^ h^−1^ under 1 sun irradiation) with low water evaporation enthalpy (841 ± 35 J g^−1^). Integration with advanced water purification techniques also demonstrates their effectiveness in removing contaminants. This strategy mimics the microstructure of wood through unidirectional freeze‐drying and offers a sustainable pathway to biodegradable solar steam generators, potentially alleviating the global water scarcity within the carbon neutrality framework.

## Graphical abstract

High efficiency solar steam generators are prepared from hydrothermal treatment of cellulose nanocrystal aerogels as an in‐situ synthesis strategy of solar absorber. They feature a biomimetic unidirectional porous structure from freeze‐drying, with low water evaporation enthalpy, and excellent solar‐driven water purification performance. This novel strategy expands the application of sustainable materials in renewable energy utilization and clean water regeneration.

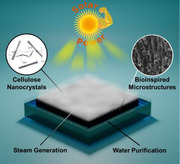

## 1 Introduction

With the growth of the world's population, rapid industrial development in many countries, and increasing frequency of droughts worldwide, the scarcity of clean water has become a serious global challenge.^[^ ^1^, ^2^ ^]^ Water scarcity affects billions of people, who are facing inadequate freshwater access and struggling to meet their basic water needs.^[^ ^3^, ^4^ ^]^ This escalating crisis calls for innovative and sustainable solutions to guarantee access to clean water. In order to address the worldwide water scarcity, various strategies have been explored, including rainwater harvesting,^[^ ^5^ ^]^ wastewater treatment and recycling,^[^ ^6^, ^7^ ^]^ desalination,^[^ ^8^ ^]^ and more.^[^ ^9^ ^]^ However, most of these methods consume large amounts of energy, so solutions that are sustainable and environmentally friendly are still urgently needed. Among the array of strategies to tackle water scarcity, solar steam generation has emerged as a particularly promising solution.^[^ ^10^ ^]^ As a sustainable technology, it operates without the consumption of electricity or fossil fuels, thus minimizing greenhouse gas emissions and mitigating environmental impacts.^[^ ^11^ ^]^ By harnessing abundant and renewable solar energy to generate steam, which can be collected as pure water, this approach offers a greener pathway to address the global water scarcity challenges compared to most other methods.

Solar absorbers in the steam generators can be classified into three main categories: noble metal nanoparticles,^[^ ^12^, ^13^ ^]^ semiconductors,^[^ ^14^, ^15^ ^]^ and carbon‐based materials.^[^ ^16^, ^17^ ^]^ Among these, carbon‐based materials including graphene,^[^ ^18^ ^]^ graphite,^[^ ^19^ ^]^ and carbon nanotubes,^[^ ^20^ ^]^ have high spectral absorption capacity and excellent light‐to‐heat conversion efficiency. Therefore, they overcome the disadvantages of the other photothermal materials, and have recently received widespread attention. However, most of these solar absorbers require a multi‐step or multi‐cycle strategy to be doped into or coated onto the hydrophilic substrates, a process that is both energy‐ and time‐consuming.^[^ ^21^, ^22^ ^]^ Moreover, the preparation of the carbon materials frequently involves the use of hazardous chemicals. Therefore, developing sustainable carbon‐based solar steam generators with high water evaporation efficiency that can also be readily prepared may revolutionize the way we approach clean water on a global scale.

Cellulose nanocrystals (CNCs) are rod‐shaped nanoparticles that can be extracted from biomass (e.g., wood pulp and cotton) using acid hydrolysis.^[^ ^23^, ^24^, ^25^ ^]^ Owing to their biocompatibility, biodegradability and accessibility from renewable resources, CNCs have been investigated as a sustainable candidate to fabricate eco‐friendly materials, including films^[^ ^26^, ^27^ ^]^ and gels.^[^ ^28^, ^29^ ^]^ Given their excellent porosity, mechanical strength and hydrophilicity, CNC‐based aerogels have been applied in solar steam generation.^[^ ^30^, ^31^ ^]^ Specifically, CNCs were used to fabricate biomimetic solar steam generators to replace unscalable sustainable materials, such as corncob pith,^[^ ^32^ ^]^ as they contain abundant surface hydroxyl functional groups, which can effectively reduce the water evaporation enthalpy. Along these lines, our group recently reported a series of CNC – reduced graphene oxide aerogel composites for application in solar steam generation.^[^ ^33^ ^]^ However, the current state of the art in cellulose‐based solar steam generators relies on an existing porous network,^[^ ^34^ ^]^ or requires additional steps to add exogenous solar absorbers to render the composite aerogels.^[^ ^35^ ^]^ The fabrication process typically requires the use of more than one type of material (i.e., hydrophilic CNCs and hydrophobic photothermal materials), which are not conducive to practical applications.

To address these issues, in this work, hydrothermally‐treated CNC aerogels (HTCAs) were fabricated as solar steam generators using a one‐pot hydrothermal gelation strategy. The hydrothermal treatment simultaneously gelled the CNC suspension and partially carbonized the CNCs, providing a novel strategy for in‐situ synthesis of the solar absorber. Moreover, the resulting HTCAs are composed only of cellulose and its derivatives, which are sustainable, biodegradable and eco‐friendly. By regulating the hydrothermal treatment temperature, the extent of carbonization of HTCAs could be adjusted, allowing for further optimization of the solar steam generation rate. Inspired by the porous, low‐tortuosity microstructure of wood, which has straight channels along its growth direction, we incorporated interconnected straight channels into the HTCAs using a unidirectional freeze‐drying strategy. The bioinspired HTCAs show low tortuosity microstructures as revealed by both scanning electron microscopy (SEM) and X‐ray microtomography (XMT), which enhances water transport and results in a 13% improvement in water evaporation rate compared to the HTCA with a disordered structure. The optimized HTCA has a low water evaporation enthalpy of 841 ± 35 J g^−1^, with a solar steam generation rate of 1.70 kg m^−2^ h^−1^ under 1 sun irradiation. Moreover, the HTCAs also show superb anti‐salt‐fouling property and water purification performance, and are fully biodegradable. These findings demonstrate an innovative approach to make high‐efficiency solar steam generators from sustainable materials, offering a green solution to address the growing global water scarcity crisis.

## 2 Results and Discussion

### 2.1 Preparation and Structural Analysis of HTCAs

**Scheme 1:**
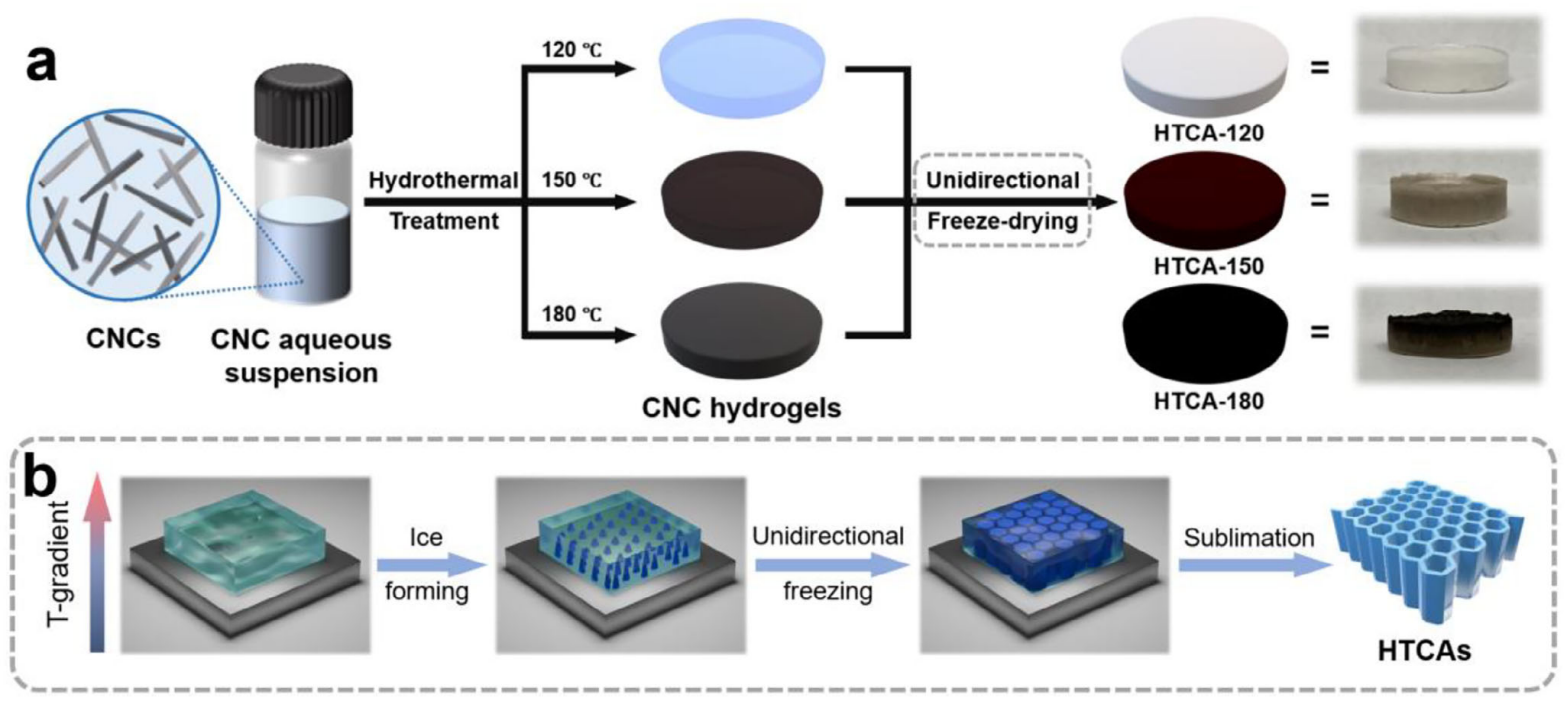
a) Materials design and standard procedure for the preparation of HTCAs. Photographs of the HTCAs are shown on the right side. b) Illustration of using the unidirectional freeze‐drying strategy to prepare HTCAs with straight channels.

**Figure 1:**
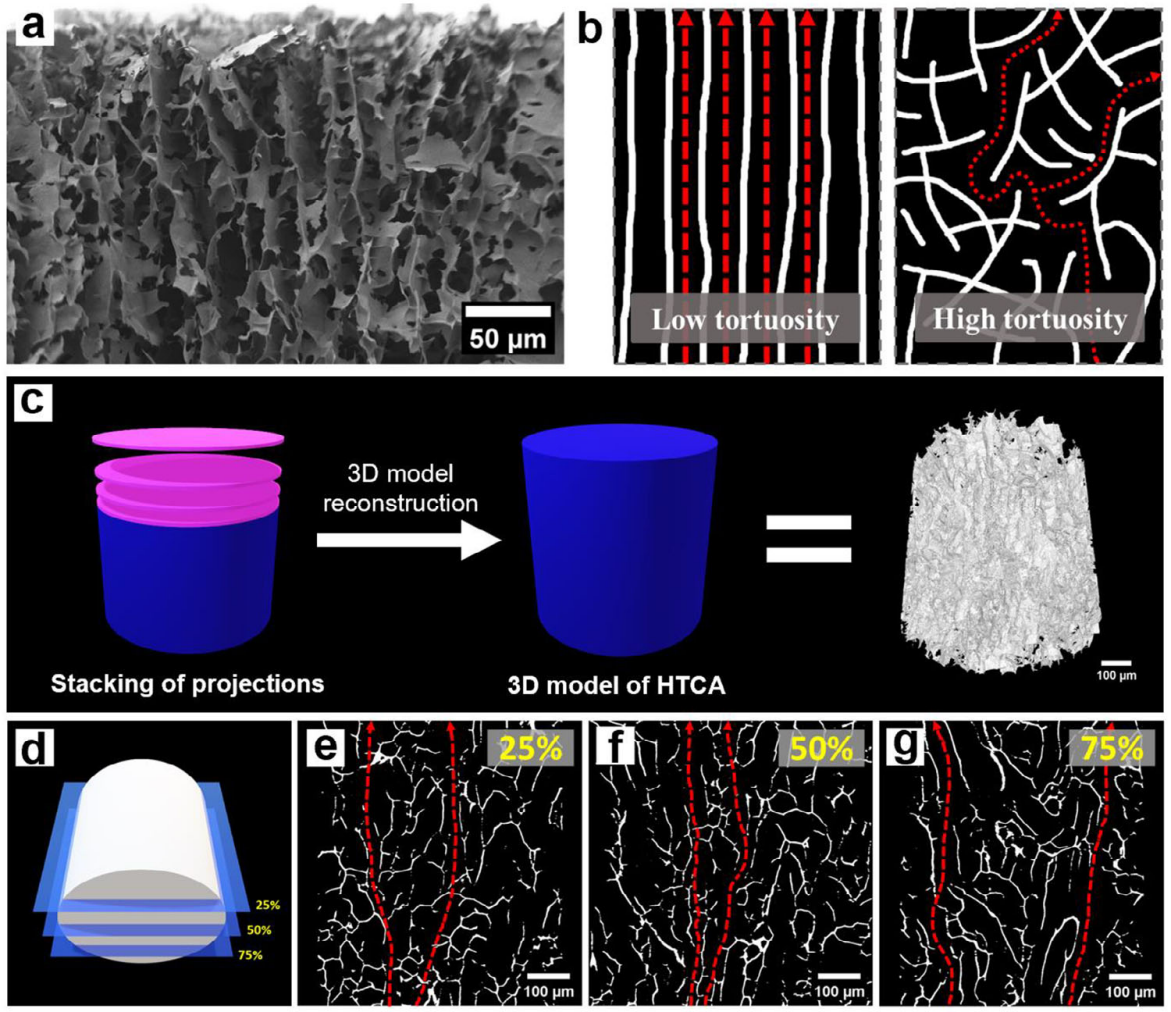
a) SEM image of the cross section of HTCA‐180 near the surface. b) Schematic illustration of water transport pathways in low tortuosity and high tortuosity microstructures. c) 3D model of HTCA‐180 assembled from the projections collected from the XMT analysis. d) Positions of resliced 2D cross‐sections in the 3D cartoon model. Cross‐sectional XMT images of HTCA‐180 taken at e) 25%, f) 50% and g) 75% position of the resliced area. The white lines correspond to CNC aerogel networks, while the black parts correspond to the channels. The red dashed arrows represent the water transport pathways.

The preparation procedure of HTCAs is illustrated in **Scheme** **1**. After performing the hydrothermal treatment and unidirectional freeze‐drying, the HTCAs were prepared as free‐standing cylindrical aerogels (Figure S2, Supporting Information). Their microstructures were revealed by scanning electron microscopy (SEM), where all samples from HTCA‐120 to HTCA‐180 show a layered structure with interconnected vertical channels originating from the unidirectional freeze‐drying (Figure S3, Supporting Information). During the freezing process, ice crystals grow from the bottom to the top, pushing the CNCs to the inter‐crystalline boundaries.^[^ ^36^ ^]^ Afterward, the ice crystals were removed by sublimation, leaving the interconnected and straight channels inside the HTCAs (Scheme 1b). Those channels are homogeneously distributed throughout the whole material, as proved by the SEM images taken at both the middle and surface regions of HTCA‐180 (**Figure** **1**a; Figure S3c, Supporting Information). Compared to the irregular pores produced by normal freeze‐drying, these ordered channels from the unidirectional freeze‐drying resemble the microstructure of wood, present lower tortuosity and provide more efficient pathways for water transport (Figure 1b). All of these are beneficial for HTCAs in solar steam generation applications.^[^ ^37^, ^38^ ^]^ Moreover, the size distributions of the channels viewed from the middle and surface regions of the HTCA‐180 cross‐sectional SEM images were determined as 20 ± 3 µm and 19 ± 3 µm, respectively (Image J). This consistency across the material demonstrates the uniformity of the unidirectional porous structure throughout the entire HTCA material.

To probe the inner microstructures of the HTCAs in more detail, HTCA‐180 was analyzed by X‐ray microtomography (XMT), a nondestructive imaging method. Figure 1c shows the reconstructed 3D cylindrical model from the 2D projections of the scanned area, where the straight channels can be clearly observed, with no visible cracks or defects. Reslicing of the 3D data set was performed from the front to back of the cylinder (Video S1, Supporting Information), and cross sections at the representative slicing positions of 25%, 50% and 75% are shown in Figure 1d. All three cross‐sectional XMT images show similar vertically‐aligned, interconnected porous microstructures (Figure 1e–g). These are not only consistent with our SEM observations, but also support the low tortuosity structure we proposed (Figure 1b) and, more importantly, further prove the homogeneity of this unique biomimetic microstructure from unidirectional freeze‐drying throughout the material.

### 2.2 Characterization and Properties of HTCAs

**Figure 2:**
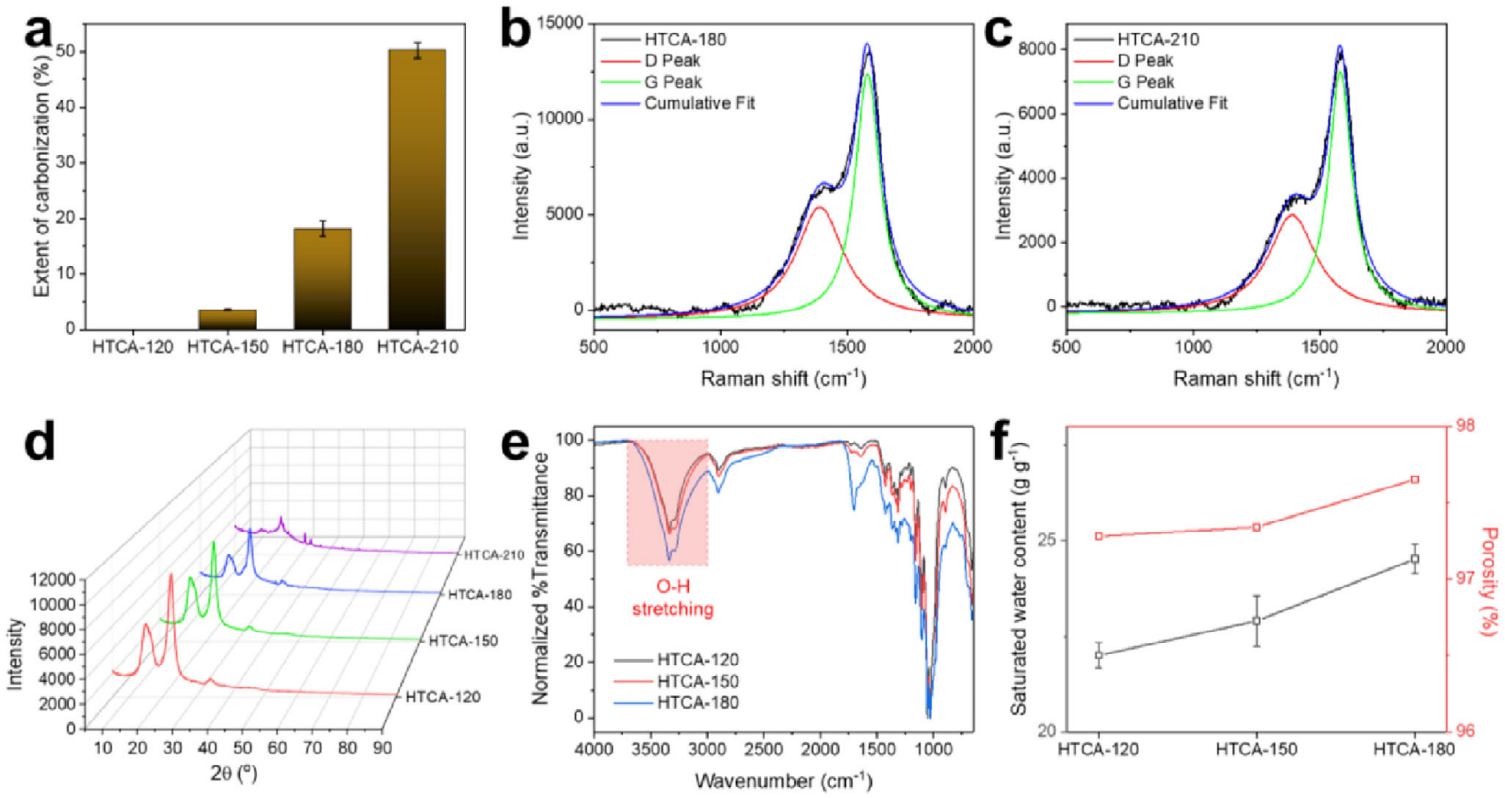
a) Extent of carbonization of HTCAs. b, c) Raman spectra of HTCA‐180 and HTCA‐210, respectively. Deconvolution and peak fitting of D and G peaks were performed using Lorentzian peak fitting in Origin 2021b, and determination of the D/G ratio was based on the maximum peak heights. d) PXRD diffractograms of HTCAs prepared under different hydrothermal temperatures. e) FTIR spectra of HTCAs. f) Saturated water content and porosity of HTCAs.

Heat localization is an important indicator that affects the performance of solar steam generators.^[^ ^39^ ^]^ By heating only a small portion of water at the air‐water interface instead of the bulk water, the water evaporation rate can be substantially increased.^[^ ^40^, ^41^ ^]^ The heat localization properties of HTCAs were determined by measuring their thermal conductivities, which decrease from 0.035 W m^−1^ K^−1^ for HTCA‐120 to 0.031 W m^−1^ K^−1^ for HTCA‐180 (Table S1, Supporting Information). This decreasing trend is concomitant with the increase of hydrothermal treatment temperature and can be explained by the increasing extent of carbonization of the HTCAs (**Figure** **2**a and Table S2, Supporting Information), which leaves less solid material available for heat transfer. This was further supported by the results of density and porosity measurements. As listed in Table S3, the density of HTCAs ranged from 0.044 g cm^−3^ to 0.038 g cm^−3^, while their porosity ranged from 97.3% to 97.7% (Figure 2f). This trend of decreasing density from HTCA‐120 to HTCA‐180 is consistent with the observed decline in their thermal conductivity. Although HTCAs present high porosity and low density, they also show high mechanical strength, with Young's moduli ranging from 0.44 to 0.62 MPa (Figure S4, Supporting Information), suggesting their high stiffness as well as their ability to maintain the original shape during deployment and usage.

Raman spectroscopy was performed to characterize this carbonaceous material formed during the hydrothermal process. Peaks corresponding to graphitic carbon (G) and disordered graphite (D) were observed at ≈1580 and ≈1350 cm^−1^, respectively (Figure 2b,c). Moreover, HTCA‐210 shows a lower D/G ratio (0.41) compared to HTCA‐180 (0.46), indicating slightly enhanced graphitization of CNCs at the higher temperature.^[^ ^42^ ^]^

PXRD diffractograms also indicate that the crystallinity of CNCs in the HTCAs decreases as the hydrothermal treatment temperature increases, as the intensity of the characteristic CNC peaks at 16.5°, 22.5°, and 34.6° decreased when the treatment temperature increased (Figure 2d). New sharp peaks observed in the pattern for HTCA‐210 were indexed to sodium sulfate (Figure S5, Supporting Information),^[^ ^43^ ^]^ confirming the desulfation of the sulfate half‐ester groups on CNCs under elevated hydrothermal treatment temperature.

Additionally, the O‐H stretching mode is detected in all HTCAs by Fourier transform infrared (FTIR) spectroscopy (Figure 2e), proving the hydroxyl groups are largely retained even after the hydrothermal treatment. An increase in saturated water content (Qs) is also observed along with the elevation of preparation temperature, ranging from 22.0 g g^−1^ for HTCA‐120 to 24.5 g g^−1^ for HTCA‐180 (Figure 2f), indicating their high hydrophilicity. The water transport rate of HTCA‐180 was approximately 14 cm/min, as proved by the observation that the 1.9 cm high HTCA‐180 sample was fully wetted by water in 8 s (Video S2, Supporting Information).

### 2.3 Solar Steam Generation Performance of HTCAs

The high porosity, low thermal conductivity, and excellent water uptake ability of HTCAs are favorable for heat localization and water transport, making them promising candidates for solar steam generation. We therefore evaluated their solar water evaporation rates using a home‐built solar evaporation testing system based on a SciSun‐300 sunlight simulator (see Experimental Section for details). For each test, the irradiation intensity at the top surface of HTCA was fixed at 1000 W m^−2^ (1 sun) and evaporation rates were measured after stabilizing under this irradiation condition for at least 60 min.

**Figure 3:**
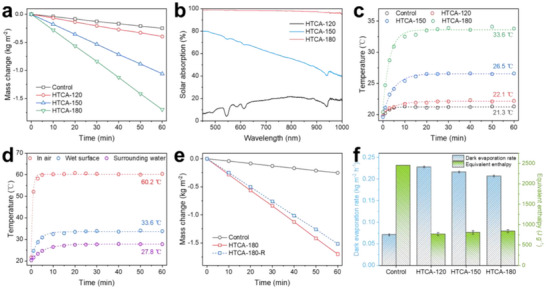
a) Mass change due to water evaporation from the surfaces of HTCAs and bulk water under one sun irradiation. b) Solar absorption spectra of the HTCAs. c) Temperature at the surfaces of bulk water and HTCAs over time under one sun irradiation. d) Surface temperature of the HTCA‐180 under air (red) and in water (blue), and its surrounding water (purple) under one sun irradiation. e) Comparison of the mass change due to water evaporation from HTCA‐180 and HTCA‐180‐R. f) The dark evaporation rate and equivalent evaporation enthalpy of bulk water and water in HTCAs, respectively.

As a control, the evaporation rate of bulk water under the experimental set up was measured as 0.25 kg m^−2^ h^−1^, consistent with previous reports.^[^ ^44^ ^]^ Three different HTCAs (HTCA‐120, HTCA‐150 and HTCA‐180) were then tested for their solar steam generation performances. After introducing the HTCAs into the bulk water, the evaporation rate was enhanced to different extents (**Figure** **3**a). The surface water evaporation rates were determined as 0.40, 1.06 and 1.70 kg m^−2^ h^−1^ for HTCA‐120, HTCA‐150 and HTCA‐180, respectively, which show 60%, 320% and 580% enhancement in evaporation efficiency, compared to that of bulk water under the same conditions. This increasing trend in solar steam generation rate can be explained by the increasing extent of carbonization of the HTCAs, which provides more solar absorbing carbonaceous material, thus enhancing the solar energy absorption and heat generation. This was further proved by their solar absorbance analysis performed using reflectance spectroscopy. As shown in Figure 3b, the HTCAs show much higher solar absorption along with the increase of their preparation temperature (i.e., extent of carbonization), and HTCA‐180 shows exceptional solar absorption ability with the average absorbance higher than 96%.

This result is further supported by the results of surface temperature monitoring during the solar evaporation process. As shown in Figure 3c, the temperature at the top surfaces of HTCAs gradually increased over the first 20 min of sunlight irradiation, and then stabilized at 22.1 °C, 26.5 °C, and 33.6 °C for HTCA‐120, HTCA‐150 and HTCA‐180, respectively. This increasing trend in stabilized surface temperature from HTCA‐120 to HTCA‐180 is in agreement with the increasing trends previously observed in their extents of carbonization and solar steam generation rates. In the case of HTCA‐120, its stabilized surface temperature remained almost the same as that of the surrounding water as well as bulk water under the same conditions (Figure S6, Supporting Information). The enhancement in water evaporation rate is mainly attributed to the formation of robust hydrogen bonds between the hydroxyl groups in HTCA‐120 and the surrounding water molecules (adsorbed water), which weakens the interactions between the absorbed water and the intermediate water from the outer layer, reducing the water evaporation enthalpy and leading to a higher evaporation rate.^[^ ^45^, ^46^ ^]^ As demonstrated for HTCA‐150 and HTCA‐180, carbonization of CNCs during the hydrothermal gelation works as an in‐situ synthesis of solar absorber, therefore leading them to much higher stabilized surface temperatures under sunlight irradiation (Figures S7, S8, Supporting Information). The higher stabilized surface temperature of HTCA‐180 in air (60.2 °C) than in water illustrates the substantial amount of thermal energy transferred from solar energy available for steam generation (Figure 3d). Furthermore, HTCA‐180 shows a 5.8 °C higher stabilized surface temperature compared to that of the surrounding water, demonstrating the high heat localization property of the HTCAs originating from their high porosity and low thermal conductivity.

The equivalent evaporation enthalpy of water in HTCAs was measured by comparing the water evaporation rates of bulk water and water‐saturated HTCAs in the dark (details see SI). Shown in Figure 3f, the calculated evaporation enthalpy of water in HTCA‐120, HTCA‐150 and HTCA‐180 is 765 ± 38, 806 ± 43 and 841 ± 35 J g^−1^, respectively. This increasing trend in water evaporation enthalpy along with the elevation of hydrothermal treatment temperature can be explained by the increasing extent of carbonization going from HTCA‐120 to HTCA‐180. This results in fewer surface hydroxyl groups from CNCs to interact with surrounding water molecules, thus reducing the evaporation enthalpy to a less extent from that of the bulk water (2450 J g^−1^). Although the water evaporation enthalpy of HTCA‐180 is slightly higher compared to the other HTCAs, all of these three values are much lower compared to most other reported solar steam generators,^[^ ^32^, ^47^ ^]^ thanks to their abundant hydroxyl groups. Moreover, its solar steam generation rate (1.70 kg m^−2^ h^−1^) is also superior compared to other previously reported carbonized materials (**Table** **1**), suggesting the great potential for HTCAs as high efficiency solar steam generators.

**Table 1:** Water evaporation rates of previously reported solar steam generators based on carbonized materials.

Carbonized material	Evaporation rate [kg m^−2^ h^−1^]	Refs.
Natural wood	1.08	[48]
Natural wood	1.21	[49]
Corncob	1.66	[50]
Pulp foam	1.62	[51]
Sunflower head	1.51	[52]
Lotus seedpod	1.30	[53]
Mushroom	1.48	[54]
Daikon	1.57	[55]
Rice husk foam	1.03	[56]
Tofu	1.65	[57]

The benefits of using unidirectional freeze‐drying during the preparation of HTCAs have been investigated through structural analyses, but have not yet been quantified. Therefore, we prepared a randomly freeze‐dried HTCA‐180 sample (HTCA‐180‐R) as a control to make a comparison with the performance of HTCA‐180. In this case, the hydrogel precursor of HTCA‐180 was directly submerged into liquid nitrogen without using a metal block to create a temperature gradient, leading to non‐directional ice crystallization. As shown in Figure 3e, HTCA‐180‐R presents a solar steam generation rate of 1.51 kg m^−2^ h^−1^. This reduced performance is likely due to the randomly ordered channels with different sizes and directions, as well as the limited interconnectivity of water pathways owing to the random growth of ice crystals,^[^ ^58^ ^]^ as evidenced by SEM analysis focusing on cross‐sectional areas (Figure S10, Supporting Information). HTCA‐180 with uniform straight channels from unidirectional freeze‐drying shows a 13% enhancement over HTCA‐180‐R, with no significant decrease in photothermal property (Figure S9, Supporting Information), illustrating the advantage of applying the unidirectional freeze‐drying strategy to prepare high efficiency solar steam generators that mimic the microstructure of wood.

Outdoor experiments were also performed to determine the enhancement in steam generation rate by using HTCAs. Under the same natural sunlight irradiation, after 10 min, the system containing HTCA‐180 started to form visible water droplets on the cap, while the control system (water only) only turns slightly foggy. After 60 min, the water droplet formed in the HTCA‐containing system is also much larger than the control system, illustrating the increase in steam generation rate after using HTCAs (Figure S11, Supporting Information). Moreover, HTCAs with lower CNC content were also systematically prepared and characterized (for details see S2.8 and S2.9, Supporting Information). However, they all show lower solar steam generation rate compared to HTCAs mentioned above.

### 2.4 Water Desalination and Purification Performance of HTCAs

**Figure 4:**
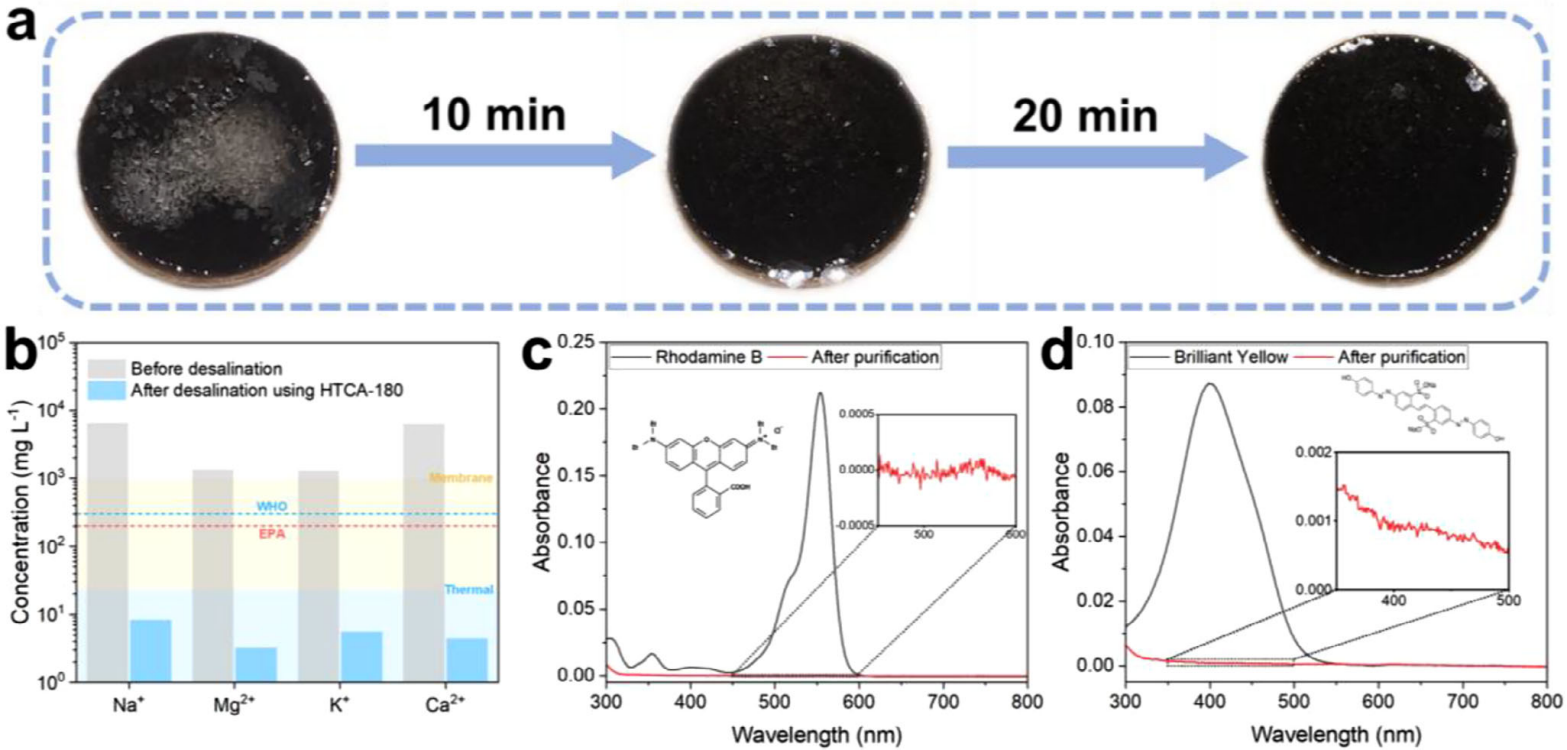
a) Photos taken at 0 min, 10 min and 30 min after adding NaCl on top of the HTCA‐180. b) Measured concentrations of four primary ions in raw seawater from Sunset Beach (Vancouver, Canada) before and after desalination using HTCA‐180. c,d) The UV–vis spectra of Rhodamine B and Brilliant Yellow simulated organic waste water after 10^4^ times dilution (black line) and after solar water purification with HTCA‐180 (red line), respectively. Insets: chemical structure of the corresponding dye molecule, and zoomed‐in UV‐vis spectra of the raw water after purification.

Solar steam generators are widely used in the area of seawater desalination and sewage purification. During steam generation, non‐volatile salts and pollutants are leftover, while the steam can be collected as freshwater. Therefore, it is essential to evaluate the effectiveness of the HTCAs in these applications. Inductively coupled plasma mass spectrometry (ICP‐MS) was used to measure the concentrations of the primary ions (Na^+^, K^+^, Ca^2+^, Mg^2+^) in raw seawater samples (obtained from Sunset Beach, Vancouver) before and after the solar desalination using HTCA‐180 (**Figure** **4**b). After undergoing desalination treatment with HTCAs, the concentrations of all four tested ions were reduced by three orders of magnitude. This reduction is comparable to other reports based on thermal and membrane desalination.^[^ ^45^ ^]^ Furthermore, the salinity levels obtained after desalination treatments are significantly lower than the drinking water standards set by the World Health Organization and the US Environmental Protection Agency.^[^ ^59^ ^]^ These results demonstrate the excellent desalination performance of the HTCAs and their great potential in water purification through solar steam generation.

During the process of desalinating seawater through solar evaporation, salt crystals may accumulate within the channels of the material, obstructing the flow of water and disrupting the evaporation of water from the surface.^[^ ^60^ ^]^ In order to determine whether salt‐fouling would be an issue for the HTCAs, sodium chloride (100 mg) was loaded on the top of HTCA‐180 under 1 sun irradiation. Figure 4a shows that the salts disappeared within 10 minutes of the solar water evaporation process. This result is comparable to other reported solar steam generation systems.^[^ ^61^, ^62^ ^]^ More importantly, HTCAs retained their high solar steam generation rate under salty conditions. When exposed to seawater, HTCA‐180 still presents high steam generation rate of 1.65 kg m^−2^ h^−1^ under 1 sun irradiation, with no significant difference in solar absorption ability compared to its performance in pure water (Figure S18, Supporting Information). These results validate the excellent anti‐salt‐fouling property of the HTCAs, making them competitive candidates for seawater desalination and water treatment applications.

Moreover, the water purification capability of HTCAs was also demonstrated by conducting the solar steam generation using 10 mg mL^−1^ dye solutions to simulate contaminated water. Rhodamine B and Brilliant Yellow were selected as representative cationic and anionic dyes, respectively. It is noteworthy that the originally contaminated water became colorless after the solar water purification process (Figure S19, Supporting Information). As shown in Figure 4c,d, the UV–vis spectra also show that the contaminated waters still absorb strongly at 555 and 400 nm, respectively, after 10^4^ times dilution. However, after solar water purification using HTCA‐180, these peaks disappeared, indicating that the water contained <10^−6 ^mg/mL Rhodamine B and <10^−5 ^mg mL^−1^ Brilliant Yellow, respectively. These results show that the HTCAs have excellent purification abilities against both positively and negatively charged water contaminants.

### 2.5 Biodegradability and Environmental Stability of HTCAs

As demonstrated in the previous sections, the whole solar steam generation system can be realized on a carbonized CNC aerogel. To prove the concept of a biodegradable solar steam generator, HTCA‐180 was subjected to a lab‐scale soil burial test. Briefly, naturally collected soil was placed in an empty glass container, and then the material's biodegradability was determined by burying it in a hole within the soil with a diameter of 20 mm and a depth of ca. 5 mm (Figure S20a, Supporting Information). During this process, the system was stored under ambient conditions (room temperature at ≈25 °C and ≈65% relative humidity), and water was added regularly to keep the soil wet. After a total of 8 weeks, the aerogel fully disappeared due to the microorganism degradation of the cellulose (Figure S20b, Supporting Information), demonstrating the fully biodegradable characteristic of the HTCAs, which is beneficial for reducing environmental impacts and realizing carbon neutrality.

Despite their high degree of biodegradability, HTCAs show good environmental stability. In an environmental stability test, HTCA‐180 was soaked in seawater for 24 hours. Subsequently, accelerated aging was induced by subjecting the system to a temperature of 90 °C. After the test, a faint brown coloration was observed in the seawater, which is indicative of carbonized materials. The physical appearance of HTCA‐180 remained unchanged, with no structural damage detected (Figure S21, Supporting Information). These results demonstrate that HTCAs have long‐term stability in seawater and can resist the accumulation of significant heat under extreme conditions in real‐life scenarios.

## 3 Conclusion

A series of hydrothermally‐treated CNC aerogels (HTCAs) was prepared using a one‐pot hydrothermal treatment followed by unidirectional freeze‐drying, a biomimetic approach to imitate the porous, low‐tortuosity microstructure of natural wood. The hydrothermal treatment not only gelled the CNC suspension, but also partially carbonized the CNCs, leading to the in‐situ formation of the graphitic solar absorber. The high hydrophilicity of CNCs and the low tortuosity of the microstructure from the unidirectional freeze‐drying enable efficient water transport inside the HTCAs. The extent of carbonization of CNCs was controlled by varying the hydrothermal temperature and CNC content to optimize the solar steam generation performance of HTCAs, showing a water evaporation rate of 1.70 kg m^−2^ h^−1^ under 1 sun irradiation with a low water evaporation enthalpy of 841 ± 35 J g^−1^. The combination of efficient solar steam generation, excellent water purification performance and complete biodegradability of HTCAs represents a promising and sustainable solution for addressing global energy and water challenges. Our findings demonstrate the potential of applying CNC aerogels as high efficiency solar steam generators with significant implications for renewable energy utilization and clean water regeneration.

## 4 Experimental Section

### Materials

All chemicals were purchased from standard suppliers and used without further purification. CNC suspensions used in this work (CNC‐Na^+^, 4.0 wt%, pH = 6.5) were diluted from CNC suspensions obtained from CelluForce Inc. (CNC‐Na^+^, 6.1 wt%, pH = 6.5) using Milli‐Q water. When they were viewed by TEM, the CNCs appear as needle‐shaped nanocrystals (Figure S1, Supporting Information). Image J was used to manually measure their length (143 ± 62 nm). Moreover, dynamic light scattering (DLS) gave a hydrodynamic radius of 155 ± 2 nm for the particles in water.

### Characterization

Transmission electron microscopy (TEM) was performed at the UBC Bioimaging Facility, using a FEI Tecnai Spirit 120 kV transmission electron microscope.

Scanning electron microscopy (SEM) was also performed at the UBC Bioimaging Facility using a Zeiss Crossbeam XB350 CryoFIB‐SEM or a Hitachi S2600 Variable Pressure SEM. All samples were sputter‐coated with 2 nm of Pd‐Au alloy prior to imaging.

Freeze‐drying was conducted at UBC Biological Services Laboratory with a Flexi‐Dry MP freeze‐dryer (−80 °C, ≈200 mT).

Raman spectroscopy was performed on a Senterra II Raman microscope (Bruker), using a 532 nm laser at 6.25 mW, with a 10 s integration time and averaging of 10 scans.

Powder X‐ray diffraction (PXRD) patterns were collected using a Malvern‐Panalytical Empyrean 3 diffractometer operating at 45 kV and 40 mA (Cu tube, λ = 1.5418 Å) under Bragg–Brentano configuration. The whole piece of the aerogel was flattened and placed on a glass slide for each test.

Dynamic light scattering (DLS) measurements were carried out 3 times for each sample using a NanoBrook Omni particle size analyzer (Brookhaven).

Elemental analysis was performed based on a Thermo Flash 2000 Elemental Analyzer under the CNH analysis mode.

FTIR patterns were obtained from a Frontier FT‐IR Spectrometer (PerkinElmer).

Thermal conductivities were measured using a TCi Thermal Conductivity Analyzer (C‐Therm). A total of 9 independent tests under a pressure of 1 N were performed for all samples, and the results were based on the average of all 9 tests.

Mechanical properties of HTCAs were assessed by compression tests based on a 5960 Series Universal Testing System (Instron) equipped with a 2 kN load cell. The stress‐strain curves were achieved from the recorded force and displacement at a loading rate of 1 mm min^−1^ under the compression mode. The Young's moduli were calculated from the initial linear portion of the stress‐strain curves through linear regression analysis.

X‐ray microtomography (XMT) analyses were conducted with a Zeiss Xradia 520 Versa X‐ray microscope. The samples were scanned at 80 kV source voltage under 7 W power with no filter applied. Projections were collected at a pixel size of 0.7 µm and then reconstructed into a 3D data set and resliced to get the cross‐sections using ImageJ (https://imagej.net/ij/).

Reflectance spectroscopy was performed using an OceanOptics setup with the following parts: light source DH‐2000‐BAL (halogen lamp), reflection probe R400‐7‐UV–vis and spectrophotometer FLAME‐S‐XR1.

UV–vis spectra were collected using a Cary 5000 UV–vis–NIR spectrometer (Agilent) with 1 cm path length quartz cuvettes.

### Preparation of Hydrothermally Treated CNC Aerogels (HTCAs)

Typically, 1 mL of CNC‐Na^+^ aqueous suspension (4.0 wt%, pH = 6.5) was transferred into a 15 mL glass scintillation vial (18 mm diameter, 26 mm height) with its top cut off. Prior to the hydrothermal treatment to render CNC hydrogels, a brief sonication (ca. 5 s) was performed to remove air bubbles from the suspension. After the hydrothermal gelation completed, the hydrogel was removed from the autoclave together with the vial and followed by performing a unidirectional freeze‐drying to give the HTCAs. They were named as HTCA‐T, where T represents the hydrothermal treatment temperature (in °C).

### Hydrothermal Treatment

Hydrothermal treatment was performed based on a reported procedure.^[^ ^63^ ^]^ Briefly, a cut vial containing the CNC‐Na^+^ suspension (1 mL, 4.0 wt%, pH = 6.5) was placed inside a Teflon‐lined stainless‐steel autoclave. It was then sealed and heated up inside a programmable oven (Thermolyne, Type 47900) to the desired temperature (120, 150, 180 or 210 °C) over 1 h and then held at that temperature for another 20 h. The system was then set to cool down to room temperature over 20 min to complete the hydrothermal treatment.

### Unidirectional Freeze‐Drying

Unidirectional freeze‐drying was performed following a previous report.^[^ ^64^ ^]^ A metal block was first submerged into liquid nitrogen (LN_2_). After ca. 5 min, the metal block was cooled down to ‐196 °C and the system reached equilibrium. The top surface of the metal block was then exposed by removing excess LN_2_. Vials containing hydrogels were then placed on top of the metal block to initiate the unidirectional freezing from the bottom to the top. After they were completely frozen, the vials were then transferred into a Flexi‐Dry MP freeze‐dryer (−80 °C, ≈200 mT) and dried to yield the HTCAs.

### Evaluation of the Solar Evaporation Rate of HTCAs

The solar evaporation testing system was constructed using a Petri dish (39 mm diameter) on a lift and a sunlight simulator (SciSun‐300, Sciencetech, Canada). Briefly, 4 mL of DI water was added into the Petri dish to float a cylindrical HTCA with a diameter of ca. 18 mm and a thickness of ca. 2 mm (Figure S22, Supporting Information). The sunlight simulator was set at 100.0 power level with all light filters removed. The height of the platform was adjusted by the lift to achieve a simulated solar flux output of 1000 W m^−2^ (1 sun) at the top surface of the HTCA. Temperature measurement was conducted by taking IR images using a Thermal Imaging Camera (Hti) during the test. The mass of the water loss was measured by an analytical balance with 0.1 mg resolution. All evaporation rates were measured after a minimum of 60 min stabilization under 1 sun irradiation.

### Statistical Analysis

The presented evaporation enthalpy data were in form of mean ± SD (SD = standard deviation), based on data collected from 5 individual tests. The presented solar steam generation rates also averaged data collected from 5 individual tests. The SDs calculated from these data were 0.03, 0.02, and 0.11 kg m^−2^ h^−1^ for HTCA‐120, HTCA‐150, and HTCA‐180, respectively.

## Conflict of Interest

The authors declare no conflict of interest.

## Supporting information



Supporting Information

Supplementary VideoS1

Supplementary VideoS2

## Data Availability

The data that support the findings of this study are available from the corresponding author upon reasonable request.
