# Supplementary material for: Bioinspired and Biodegradable Hydrothermally Treated Cellulose Nanocrystal Aerogels for High Efficiency Solar Steam Generation and Sustainable Water Purification
Source: Small. 2025 Oct 30;21(50):e08897. doi: 10.1002/smll.202508897 (PMC12710203; doi:10.1002/smll.202508897)
Supplement: Supplementary file 1 — Supporting Information [file SMLL-21-e08897-s002.pdf]

NANO · MICRO small

Supporting Information

for *Small*, DOI 10.1002/smll.202508897

Bioinspired and Biodegradable Hydrothermally Treated Cellulose Nanocrystal Aerogels for High Efficiency Solar Steam Generation and Sustainable Water Purification

*Zongzhe Li, Stephanie Co, James G. Drummond, D. Mark Martinez, Feng Jiang and Mark J. MacLachlan**

Supporting Information

Bioinspired and Biodegradable Hydrothermally Treated Cellulose Nanocrystal Aerogels for High Efficiency Solar Steam Generation and Sustainable Water Purification

Zongzhe Li[a], Stephanie Co[a], James G. Drummond[b,c], D. Mark Martinez[b,c], Feng Jiang[d] and Mark J. MacLachlan[a,e,f,g]*

[a]Department of Chemistry, University of British Columbia, 2036 Main Mall, Vancouver, British Columbia, V6T 1Z1, Canada

[b]Department of Chemical and Biological Engineering, University of British Columbia, 2360 East Mall, Vancouver, British Columbia, V6T 1Z3, Canada

[c]Pulp and Paper Centre, University of British Columbia, 2385 East Mall, Vancouver, British Columbia, V6T 1Z4, Canada

[d]Sustainable Functional Biomaterials Laboratory, Department of Wood Science, University of British Columbia, 2424 Main Mall, Vancouver, British Columbia, V6T 1Z4, Canada

[e]Stewart Blusson Quantum Matter Institute, University of British Columbia, 2355 East Mall, Vancouver, British Columbia, V6T 1Z4, Canada

[f]WPI Nano Life Science Institute, Kanazawa University, Kanazawa, 920-1192, Japan

[g]Bioproducts Institute, University of British Columbia, 2360 East Mall, Vancouver, British Columbia, V6T 1Z3, Canada

*mmaclach@chem.ubc.ca

S1. Supporting Methods

S1.1. Calculation of the Extent of Carbonization (EOC_T) of HTCAs

The calculation of the extent of carbonization of HTCAs was based on their carbon and hydrogen contents ($W_{C,T}$ and $W_{H,T}$, both in percentage) from the elemental analysis results, where T represents the hydrothermal temperature. By assuming the extent of carbonization of HTCA-120 is 0, the extent of carbonization of other HTCAs can be calculated from the following Equation 1:

$$EOC_T = (W_{C,T} - W_{H,T}/(W_{C,120} \times W_{H,120}))/W_{C,T} \times 100 \tag{1}$$

S1.2. Calculation of the Bulk Density (ρ) and Porosity (φ) of HTCAs

The bulk density (ρ) of the cylindrical HTCAs were calculated based on the following Equation 2:

$$\rho = 4m/\pi d^2 h \tag{2}$$

where m, h and d represent their mass, height and diameter, respectively.

Following that, the porosity (φ) of HTCAs can be further calculated based on the Equation 3:

$$\varphi = (1 - \rho/\rho_{CNC}) \times 100 \tag{3}$$

where ρ and ρ_{CNC} represent the bulk density of HTCAs and the density of pure CNC, respectively.

S1.3. Calculation of the Saturated Water Content (Q_s) of HTCAs

Typically, a piece of freshly dried HTCA (~10 mg) was transferred into a pre-weighed 20 mL glass scintillation vial. The aerogel was then weighed together with the vial. After that, the aerogel was soaking into excess amount of Milli-Q water for a minimum of 15 min to guarantee the aerogel was fully saturated. The excess water was

then fully removed, and the saturated aerogel was weighed together with the vial. The calculation of the saturated water content (Q_s) is based on Equation 4:

$$Q_s = (m_3 - m_2)/(m_2 - m_1) \quad (4)$$

where the m_1, m_2 and m_3 are corresponding the mass of the empty vial, the vial with aerogel and the vial with water-saturated aerogel, respectively. A total of three separate samples were used to collect the data for each HTCA.

S1.4. Evaluation of Evaporation Enthalpy (E_e) of Water in HTCAs

By assuming the identical energy input (U_{in}) in a dark, ambient condition over 1h, and comparing the weight loss of the water-saturated HTCAs (m_e) and bulk water with the same surface area (m_0). Together with the theoretical enthalpy of liquid water (E_0, 2450 J/g), we can estimate the evaporation enthalpy of HTCAs using the following Equation 5:

$$U_{in} = E_e m_e = E_0 m_0 \quad (5)$$

Please note that during the actual solar steam generation rate tests, the HTCAs were floating in water, and the porous structure was not fully exposed to air. Therefore, the enhancements of the water evaporation rate demonstrated in this evaporation enthalpy analysis are reasonably higher than that in the solar steam generation rate tests.

S2. Supporting Figures and Tables

S2.1. Characterization of CNCs

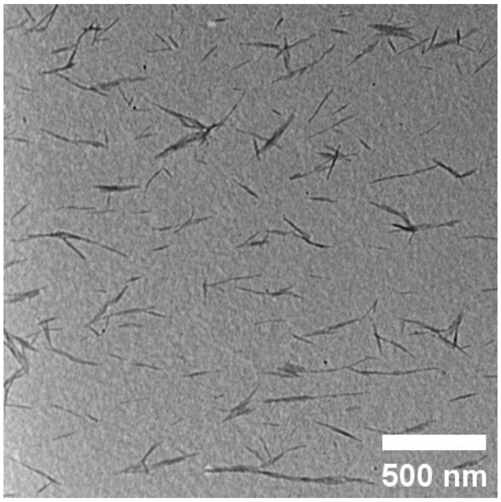

Figure S1. TEM image of CNCs. Image J (NIH, http://imagej.nih.gov/ij/) was used to manually measure the length of CNCs as 143 ± 62 nm (152 particles counted).

S2.2. Preparation of HTCAs

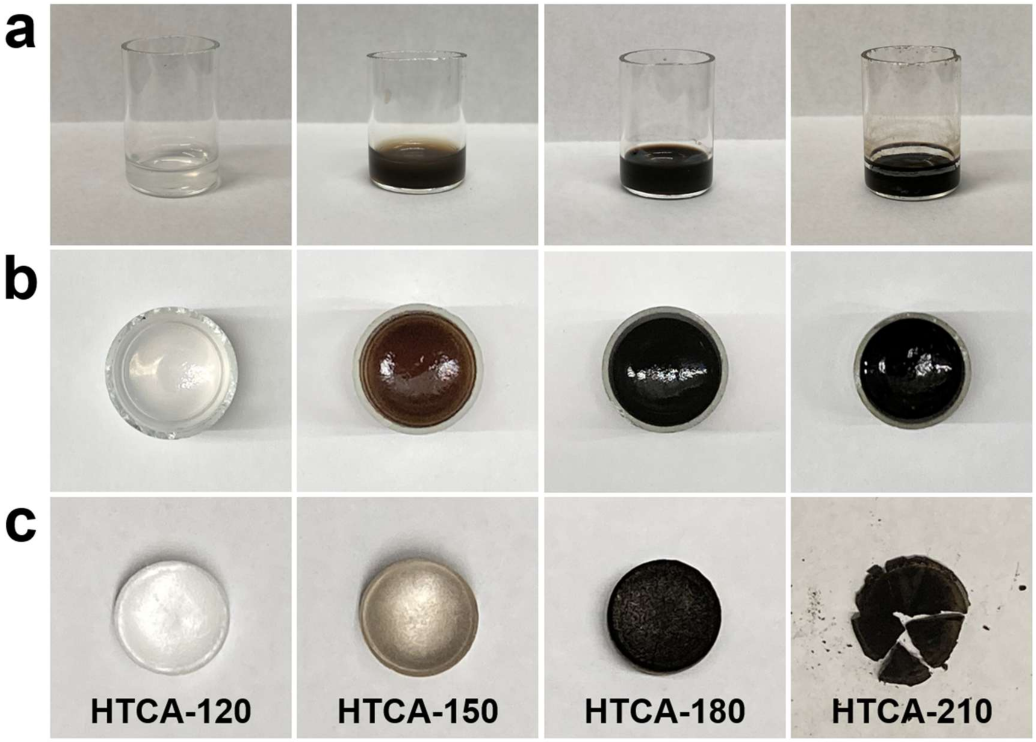

Figure S2. (a) Side view and (b) top view of the hydrogel precursors of HTCAs after the hydrothermal treatment. (c) Top view of HTCAs prepared under different hydrothermal temperatures (HTCA-120, HTCA-150, HTCA-180 and HTCA-210). The darker color of the HTCAs along with the increasing of hydrothermal temperature indicating an increasing trend of carbonization extent. Note that due to the fragile nature of HTCA-210, some tests and characterizations are not applicable to it.

S2.3. Structural Analysis of HTCAs

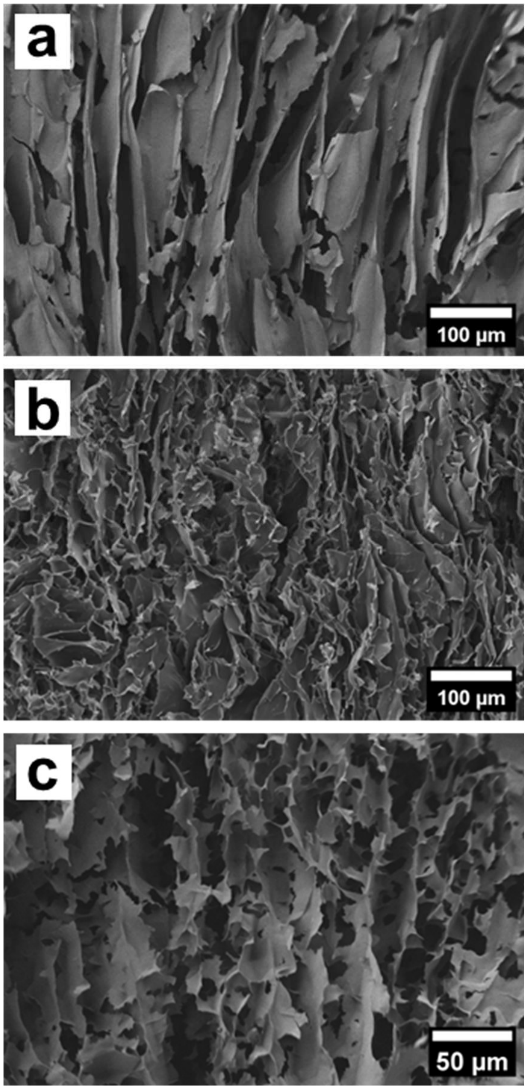

Figure S3. Cross-sectional SEM images of (a) HTCA-120, (b) HTCA-150 and (c) HTCA-180. Image J (NIH, http://imagej.nih.gov/ij/) was used to manually measure the pore size.

S2.4. Characterization of HTCAs

Table S1. Thermal conductivity of HTCAs

Sample	HTCA-120	HTCA-150	HTCA-180
Thermal Conductivity [W m^{-1} K^{-1}]	0.0348 ± 0.0004	0.0332 ± 0.0004	0.0311 ± 0.0003

Table S2. Calculated carbonization extent of HTCAs

Sample	HTCA-120	HTCA-150	HTCA-180	HTCA-210
Carbonization Extent [%]	0.0	3.6 ± 0.1	18.1 ± 1.4	50.5 ± 1.4

Table S3. Calculated density and porosity of HTCAs

Sample Name	Diameter [mm]	Thickness [mm]	Weight [mg]	Density [g cm^{-3}]	Porosity [%]
HTCA-120	18.1	3.87	43.9	0.0443	97.28
HTCA-150	18.0	3.78	42.4	0.0434	97.34
HTCA-180	17.9	3.59	34.6	0.0382	97.65

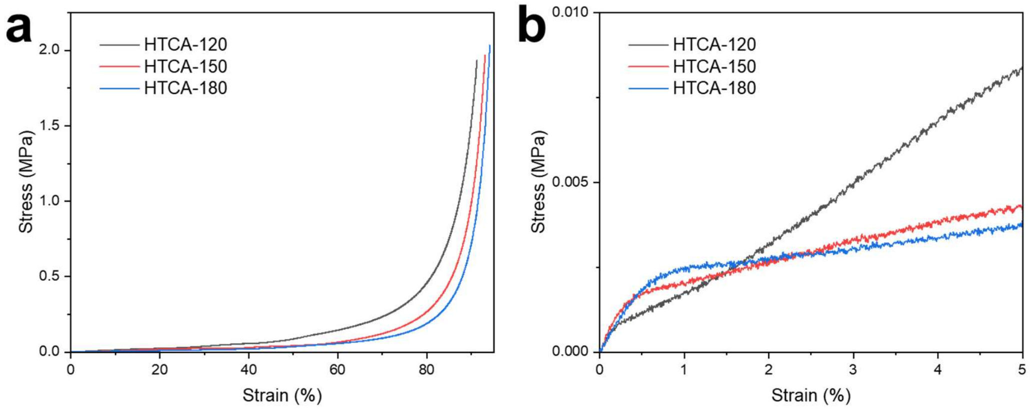

Figure S4. (a) Full and (b) partially enlarged stress-strain curves of HTCAs.

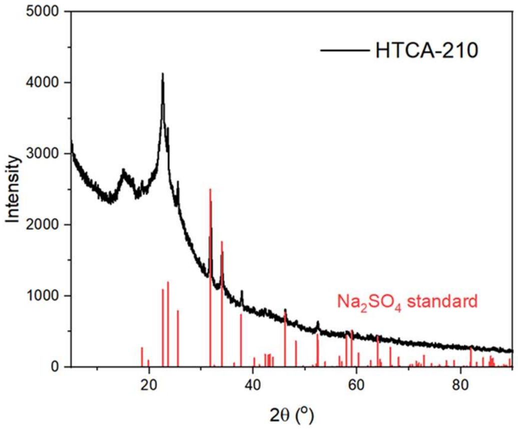

Figure S5. PXRD diffractogram of HTCA-210 compared with the standard pattern from sodium sulfate (Orthorhombic, COD: 96-210-7320).[1]

S2.5. Temperature Monitoring at the Surfaces of HTCAs During Tests

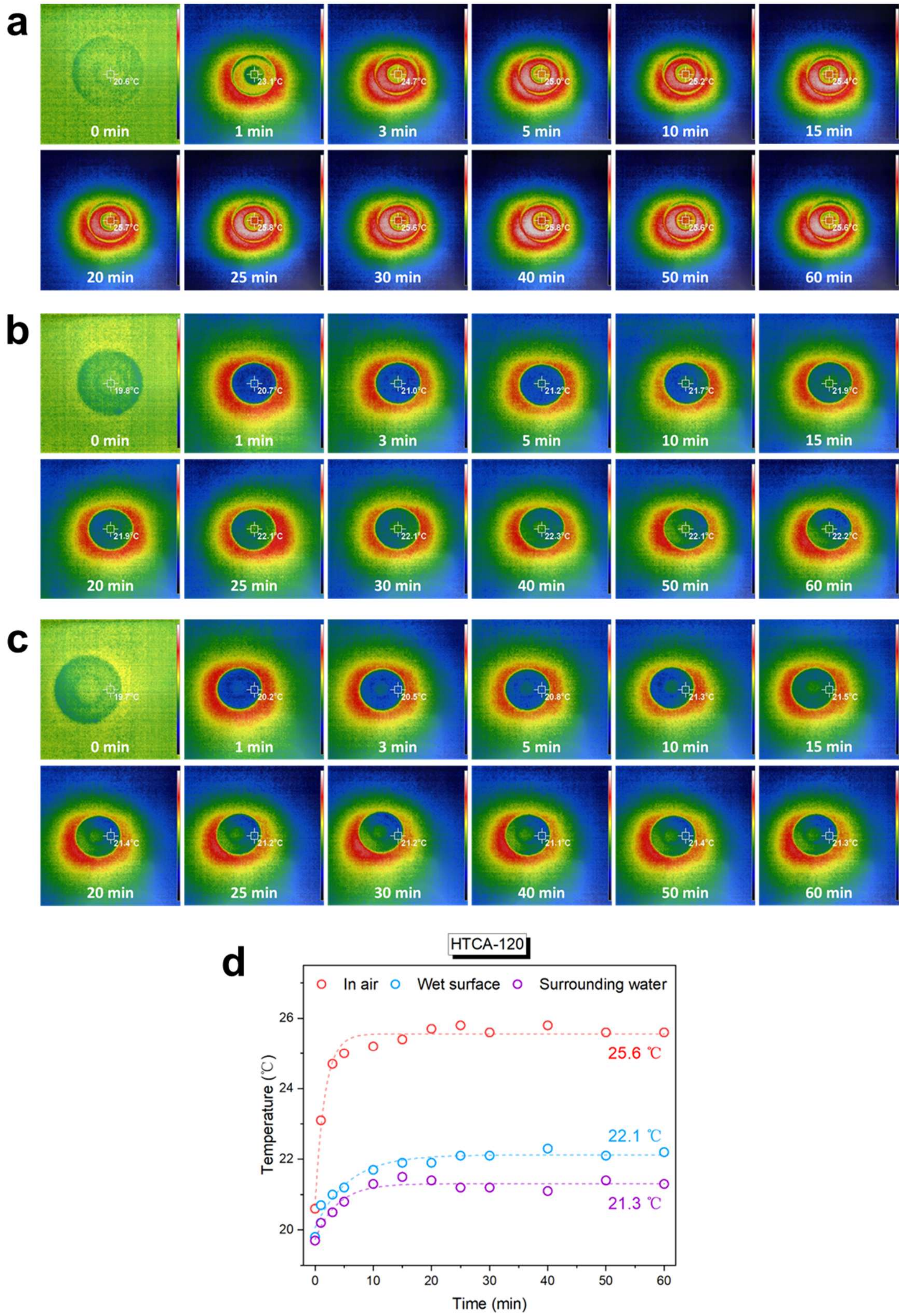

Figure S6. The infrared images to detect the surface temperature of HTCA-120 (a) in air, (b) in water, and (c) the surface temperature of its surrounding water during 60 min under one sun irradiation. (d) Surface temperature of the HTCA-120 under air (red) and in water (blue), and its surrounding water (purple) under one sun irradiation.

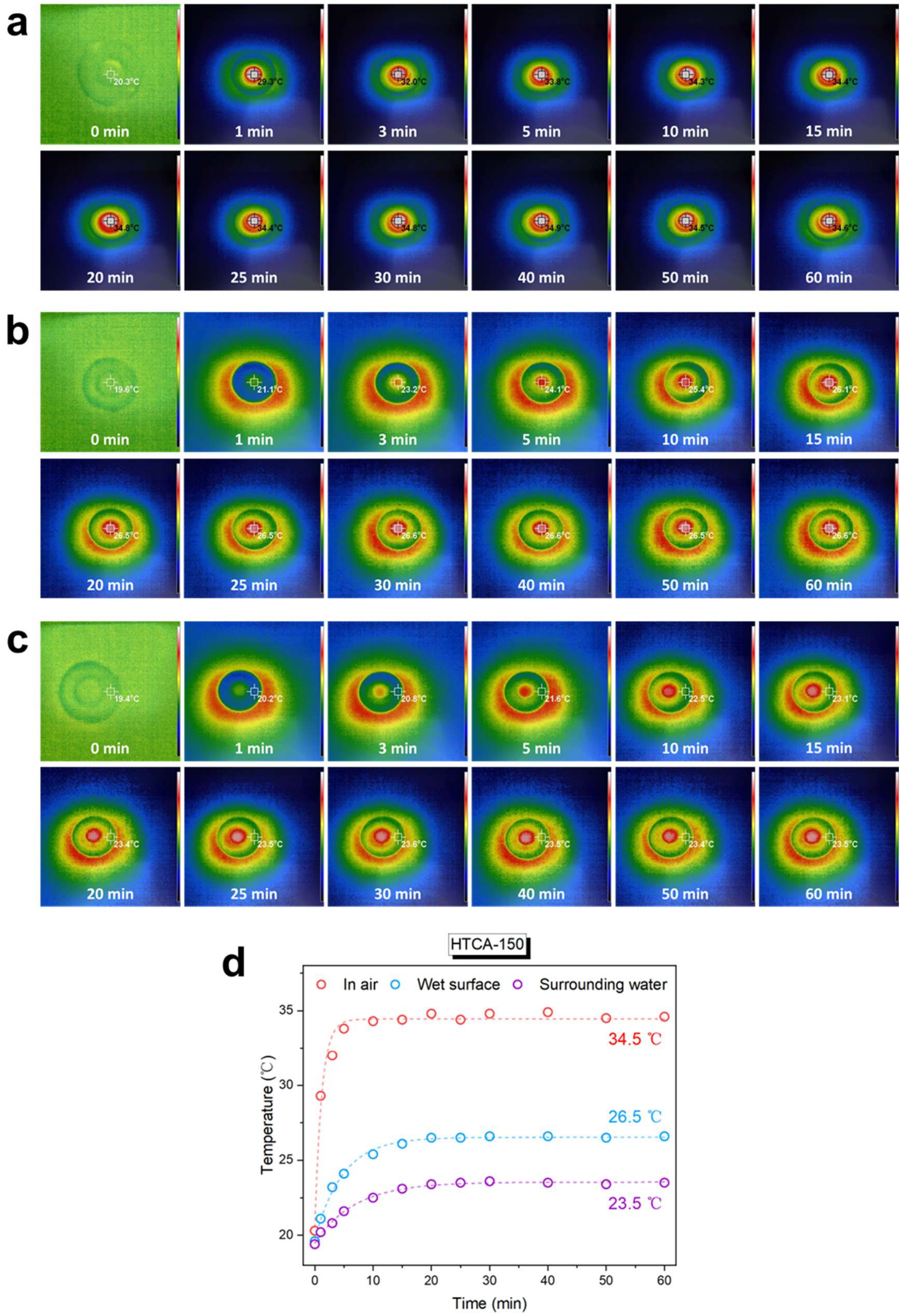

Figure S7. The infrared images to detect the surface temperature of HTCA-150 (a) in air, (b) in water, and (c) the surface temperature of its surrounding water during 60 min under one sun irradiation. (d) Surface temperature of the HTCA-150 under air (red) and in water (blue), and its surrounding water (purple) under one sun irradiation.

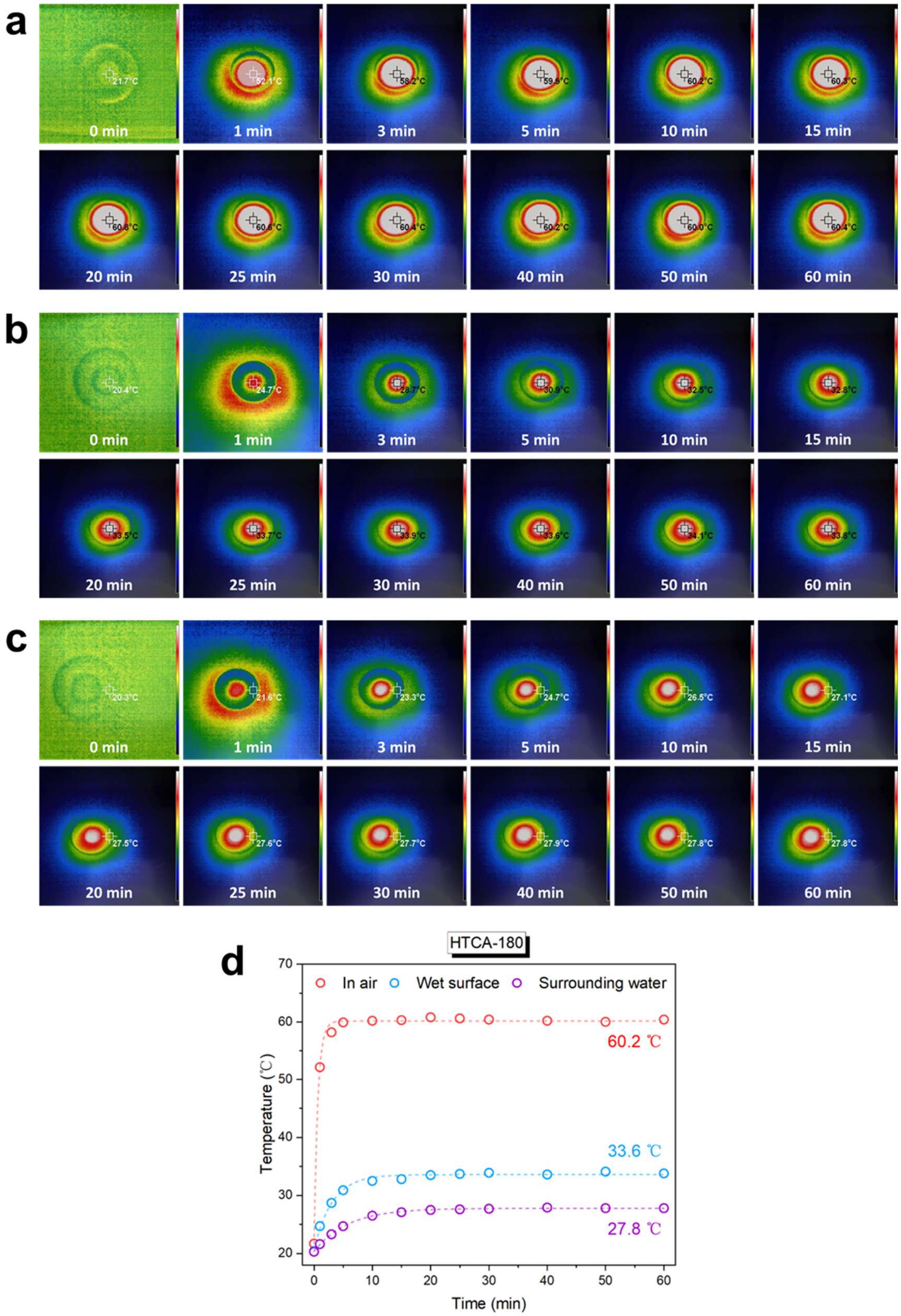

Figure S8. The infrared images to detect the surface temperature of HTCA-180 (a) in air, (b) in water, and (c) the surface temperature of its surrounding water during 60 min under one sun irradiation. (d) Surface temperature of the HTCA-180 under air (red) and in water (blue), and its surrounding water (purple) under one sun irradiation.

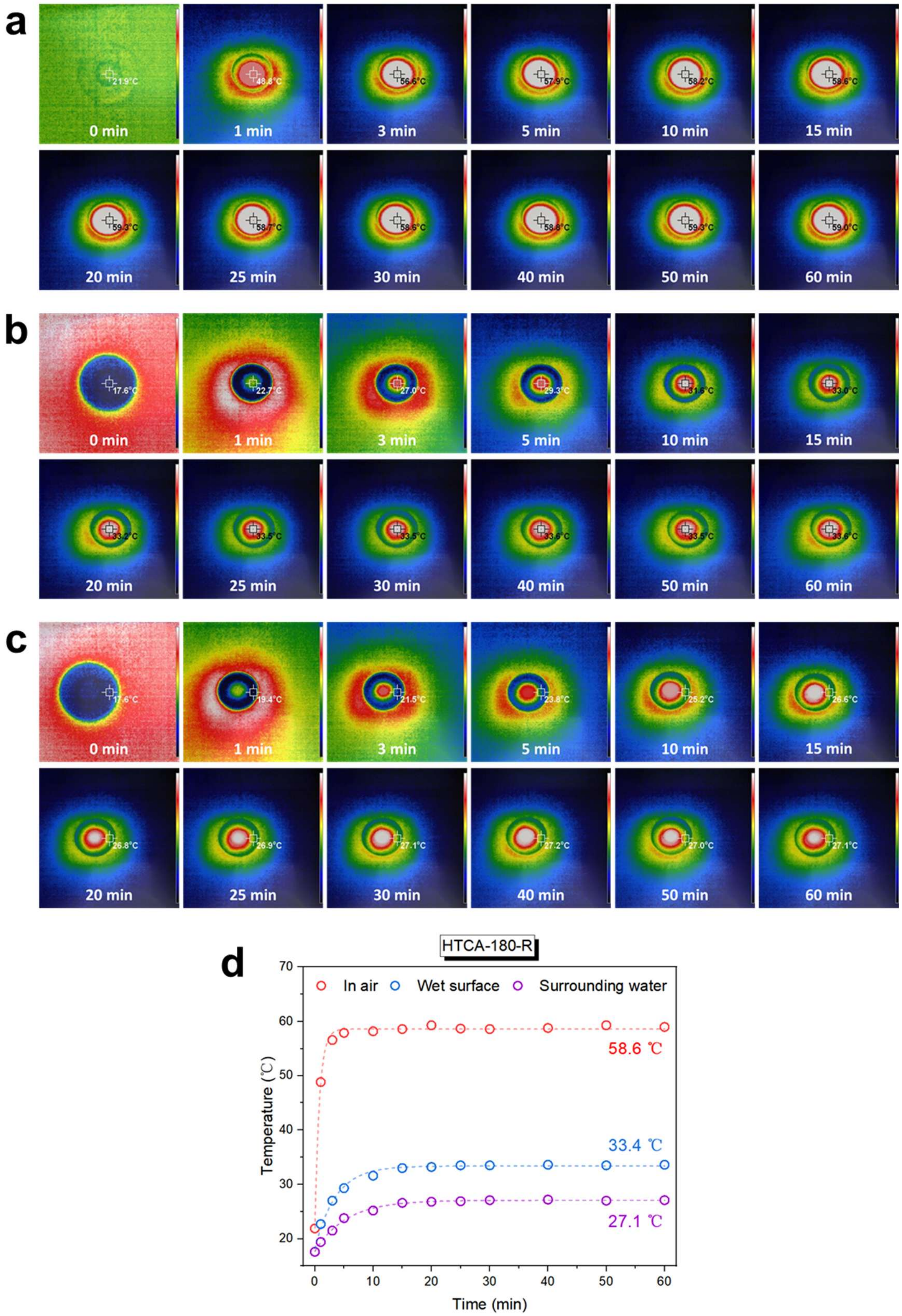

Figure S9. The infrared images to detect the surface temperature of HTCA-180-R (a) in air, (b) in water, and (c) the surface temperature of its surrounding water during 60 min under one sun irradiation. (d) Surface temperature of the HTCA-180-R under air (red) and in water (blue), and its surrounding water (purple) under one sun irradiation. Showing comparable stabilized temperatures to HTCA-180.

S2.6. Structural Analysis of HTCA-180-R

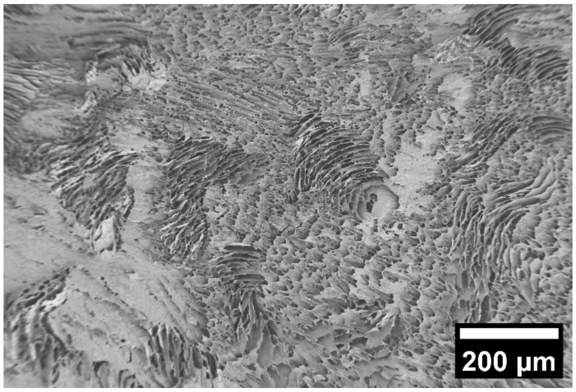

Figure S10. Cross-sectional SEM image of HTCA-180-R, where the randomly ordered pores with various sizes and pointing directions are observed in different regions.

S2.7. Outdoor Solar Steam Generation Tests

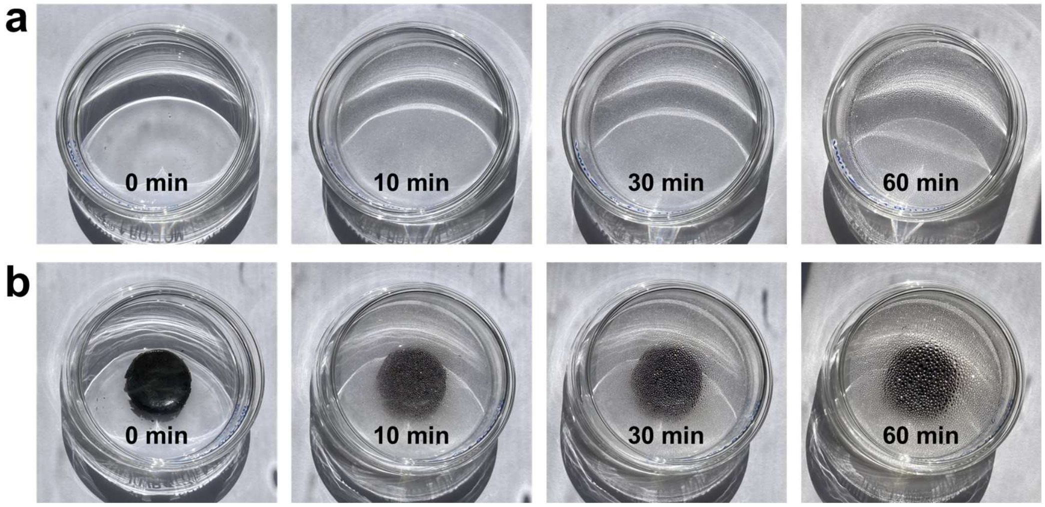

Figure S11. Solar steam generation set-up of (a) control, and (b) HTCA-180. Diameter of petri dish = 60 mm.

S2.8. Preparation of HTCAs with Lower CNC Content

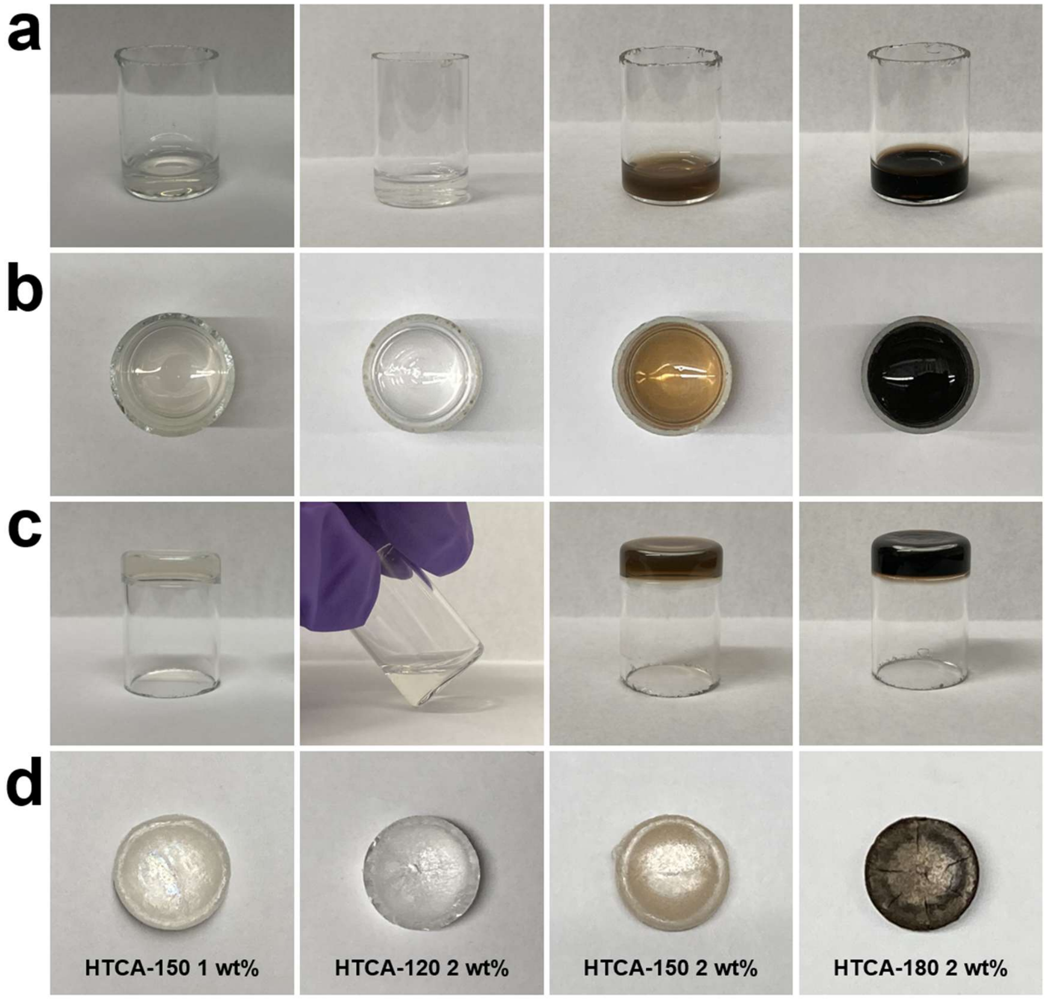

Figure S12. (a) Side view and (b) top view of the precursors of HTCAs with lower CNC content after the hydrothermal treatment. (c) Inversion tests for the HTCA precursors. Please note that the precursor of HTCA-120 2 wt% didn't pass the inversion test. (d) Top view of HTCAs with lower CNC content (HTCA-150 1 wt%, HTCA-120 2 wt%, HTCA-150 2 wt% and HTCA-180 2 wt%).

Previous reports showed that lower solid content in solar steam generators may lead to enhanced efficiency in solar steam generation.[2] We therefore explored the impact of CNC content on the solar steam generation performance of HTCAs. CNC suspensions at lower concentrations (1 and 2 wt%) were prepared by diluting the 4 wt% CNC suspension (CNC-Na^+, pH = 6.5) with Milli-Q water. The HTCA samples were prepared following the same procedure as described in the Experimental Section, but varying the concentration of the CNC suspensions being used (**Figure S12**). The

resulting materials are named as “HTCA-T X wt%”, where T represents the hydrothermal treatment temperature (in °C) and X represents the concentration of CNC suspension used, respectively.

S2.9. Performance and Properties of HTCAs with Lower CNC Content

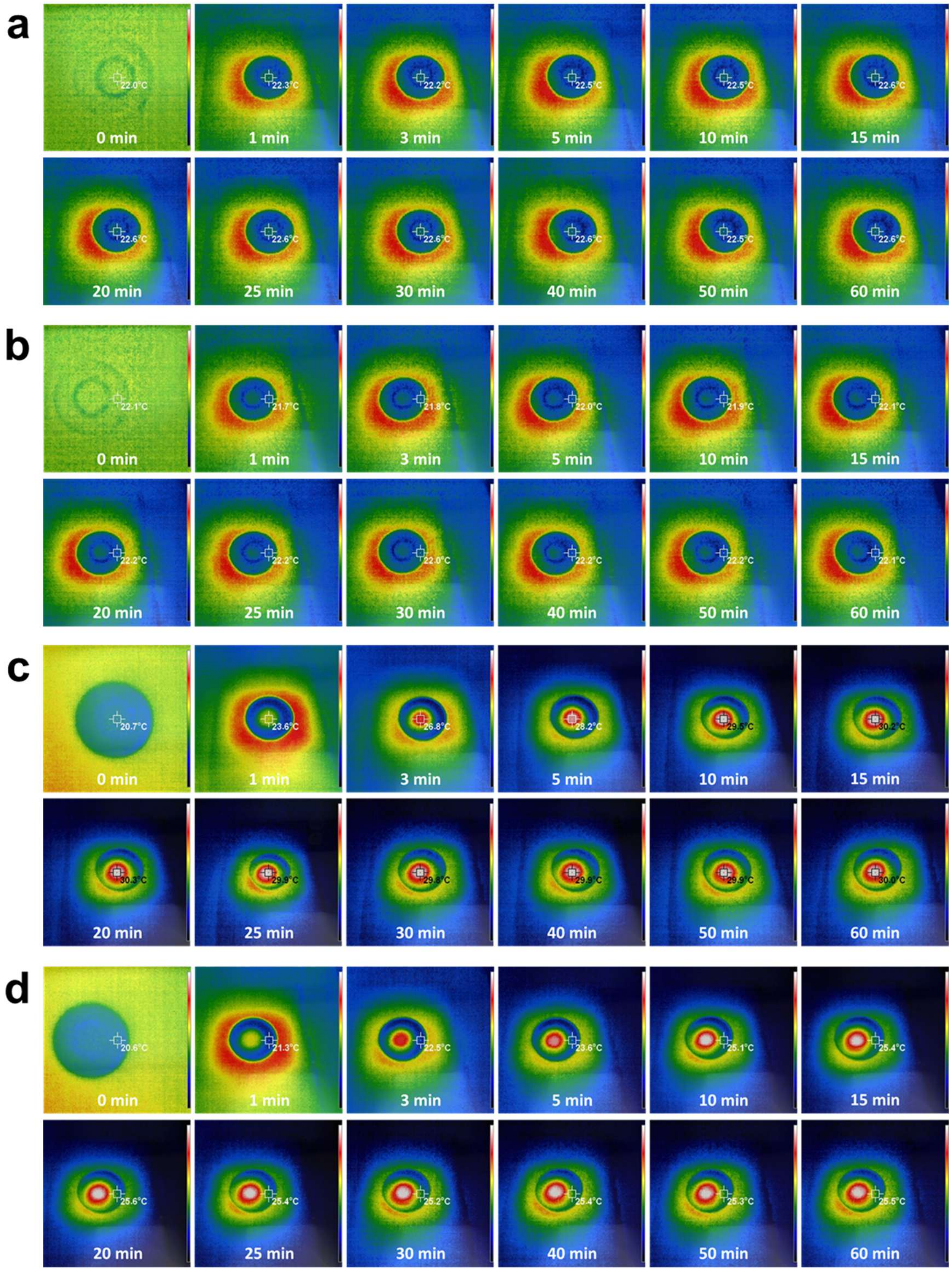

Figure S13. (a-b) Infrared images to determine the surface temperature of HTCA-150 2 wt% and its surrounding water during 60 min under one sun irradiation, respectively. (c-d) Infrared images to determine the surface temperature of HTCA-180 2 wt% and its surrounding water during 60 min under one sun irradiation, respectively.

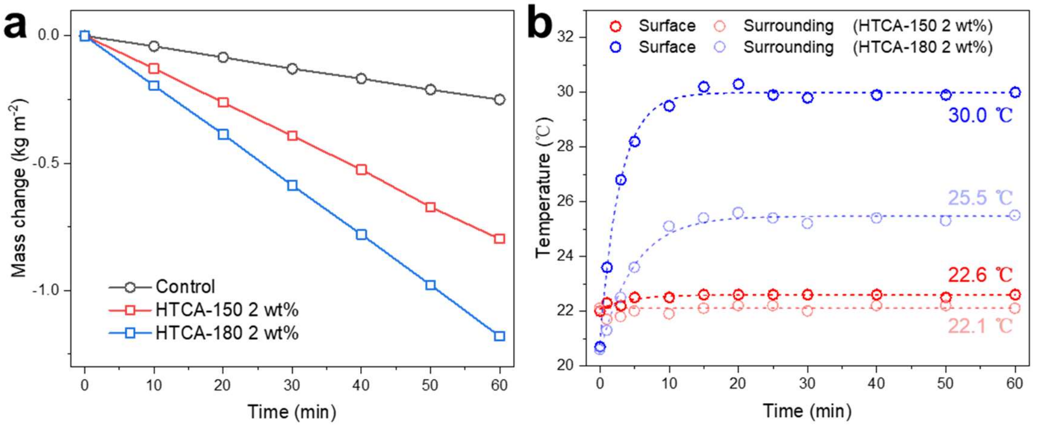

Figure S14. (a) Mass change of evaporated water from the 2 wt% HTCA surfaces and bulk water under one sun irradiation. (b) Temperatures at the surface of bulk water and 2 wt% HTCAs over time under one sun irradiation.

As shown in **Figure S14a**, HTCA-150 2 wt% and HTCA-180 2 wt% showed solar water evaporation rates of 0.80 and 1.18 kg m^{-2} h^{-1}, respectively. The surface temperature monitoring results also showed a higher stabilized surface temperature for HTCA-180 2 wt% compared to HTCA-150 2 wt% (**Figure S14b**). Both of these observations are consistent with the trend seen for the corresponding HTCA samples prepared using 4 wt% CNC suspensions (4 wt% HTCAs). That is, the higher hydrothermal treatment temperature leads to higher solar steam generation rate, as well as higher stabilized surface temperature.

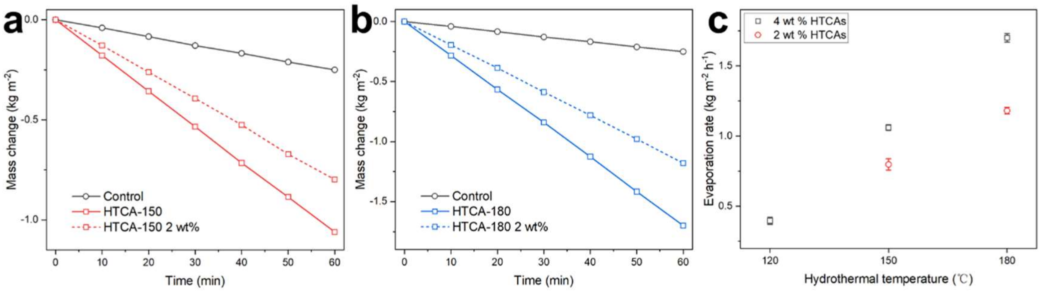

Figure S15. Comparison of the mass change between (a) HTCA-150 and HTCA-150 2 wt%, and (b) HTCA-180 and HTCA-180 2 wt%, during the solar steam generation tests. (c) Water evaporation rates of 4 wt% HTCAs and 2 wt% HTCAs under 1 sun irradiation.

Contrary to our previous expectations, HTCA-150 2 wt% and HTCA-180 2 wt% (2 wt% HTCAs) show lower solar steam generation rates compared to their corresponding

4 wt% HTCAs, with a decrease of 25% and 30%, respectively (**Figure S15**). However, this can be rationalized by their lighter colors, which may indicate a lower content of carbonized CNC as solar absorber. This was proved by calculating the extent of carbonization of HTCAs with lower CNC content based on the elemental analysis results (**Table S4**). Furthermore, the 2 wt% HTCAs also have lower stabilized surface temperatures compared to their corresponding 4 wt% HTCAs (**Figure S13** & **Figure S14b**). Please note that owing to their fragile nature, HTCA-120 2 wt% and HTCA-150 1 wt% came apart or shrank dramatically while soaking with water (**Figure S16**). Therefore, they were not used for further experiments, including tests of solar steam generation efficiency and saturated water content.

Table S4. Carbonization extent of HTCAs with lower CNC content

Sample	HTCA-150 1%	HTCA-150 2%	HTCA-180 2%
Carbonization Extent [%]	0.3 ± 0.1	1.3 ± 0.2	14.0 ± 0.9

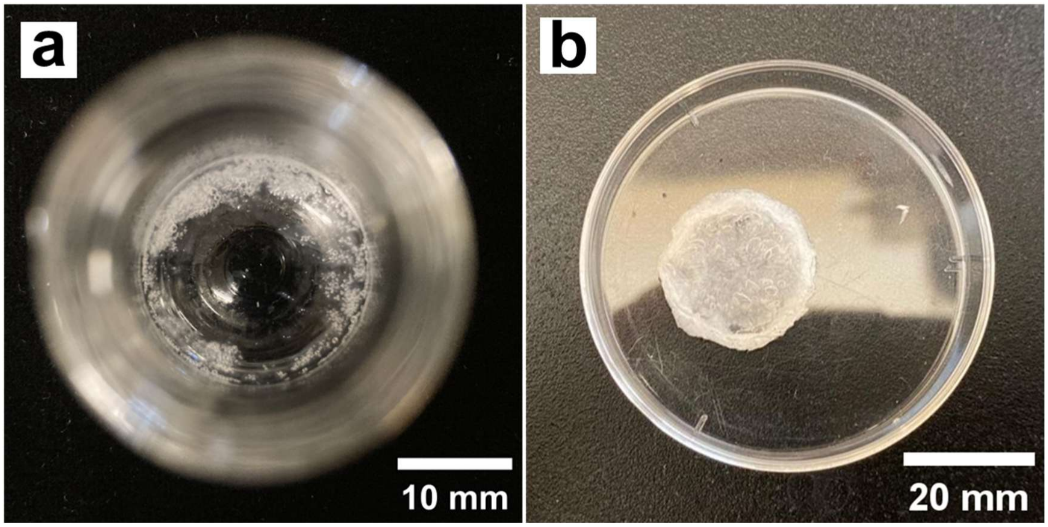

Figure S16. Physical appearance of (a) HTCA-150 1 wt% and (b) HTCA-120 2 wt% after soaking with water.

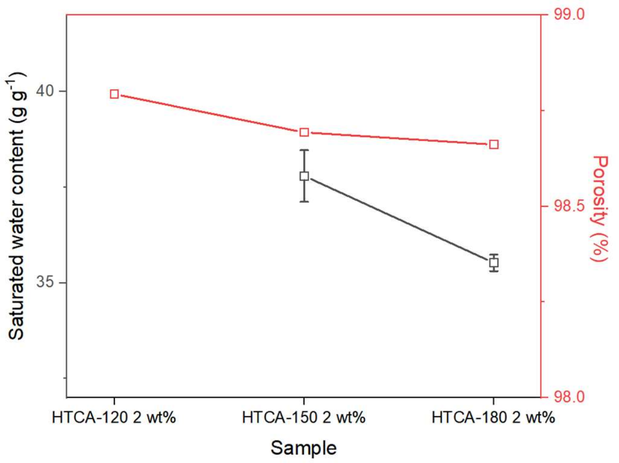

Figure S17. Saturated water content and porosity of 2 wt% HTCAs.

The porosity and Q_s of the 2 wt% HTCAs were also measured, and the results are shown in **Figure S17**. Owing to their lower solid contents, they all show higher porosity and Q_s in comparison with their corresponding 4 wt% HTCAs. However, with increased hydrothermal temperature, the 2 wt% HTCAs show a decreasing trend in porosity, in contrast with the increasing trend observed with the 4 wt% HTCAs. We speculate that the lower solid content in the 2 wt% HTCAs may be insufficient to maintain a robust 3D network during the high temperature hydrothermal treatment, therefore leading to shrinkage of the resulting materials.

S2.10. Seawater Steam Generation Performance of HTCA-180

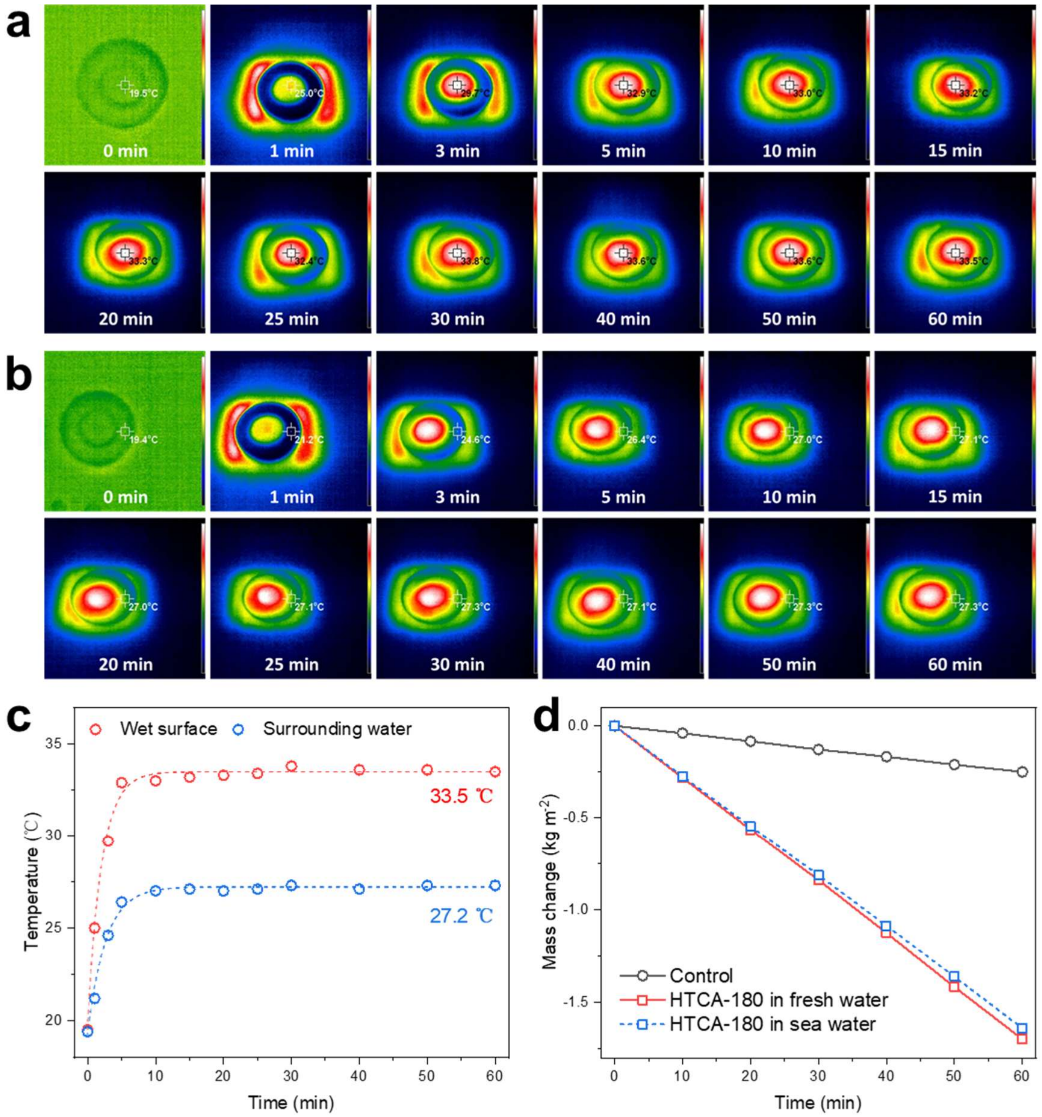

Figure S18. (a-b) Infrared images to determine the surface temperature of HTCA-180 and its surrounding seawater during 60 min under one sun irradiation, respectively. (c) Temperatures at the surface of HTCA-180 and its surrounding seawater over time under one sun irradiation. (d) Mass change of evaporated water from the bulk water surface and the HTCA-180 surface in seawater and under one sun irradiation.

S2.11. Solar Water Purification Performances of HTCAs

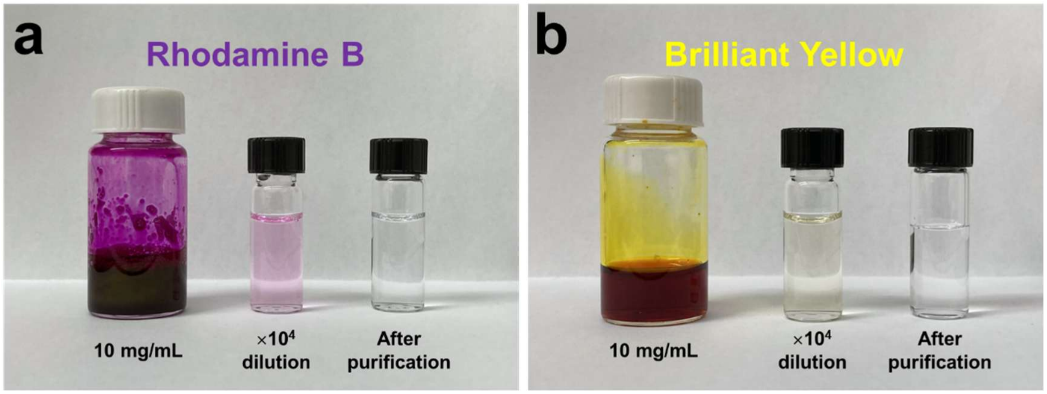

Figure S19. Physical appearance of (a) Rhodamine B and (b) Brilliant Yellow solutions: 10 mg/mL solutions used for dye removal tests; solutions diluted 10^4 times for UV-vis analysis; solutions after solar water purification.

S2.12. Biodegradability of HTCAs

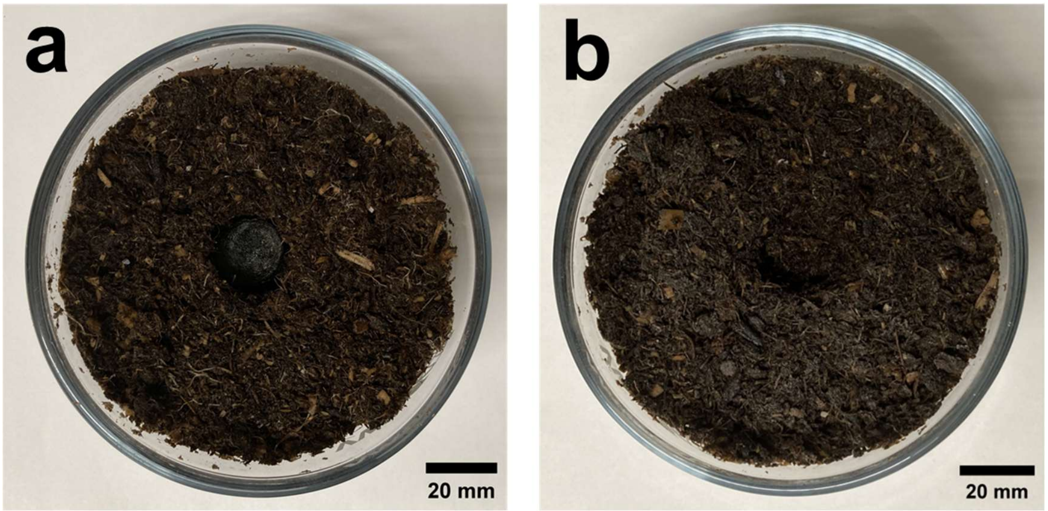

Figure S20. Biodegradation tests of HTCAs. Photographs of HTCA-180 in soil (a) right after putting inside the hole and (b) after 8 weeks.

S2.13. Environmental Stability of HTCAs

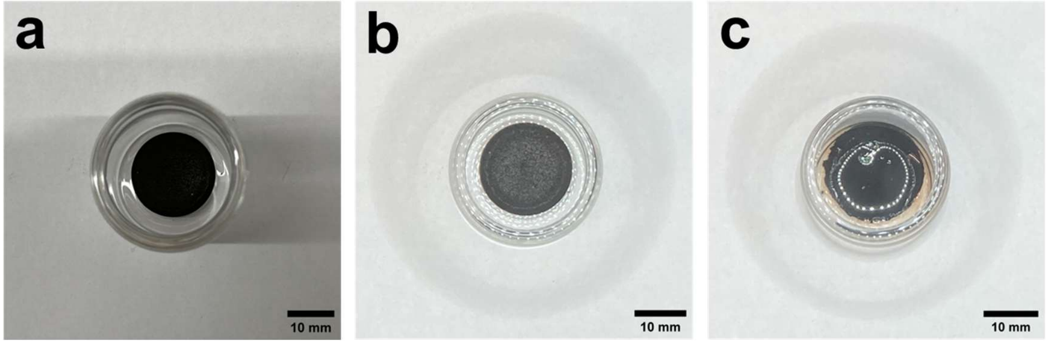

Figure S21. Accelerated environmental stability test of HTCAs. Photographs of HTCA-180 (a) placed in a 20 mL vial, (b) after adding water, and (c) after soaking in 90 ℃ water for 24 h.

S2.14. Construction of the Solar Steam Generation System

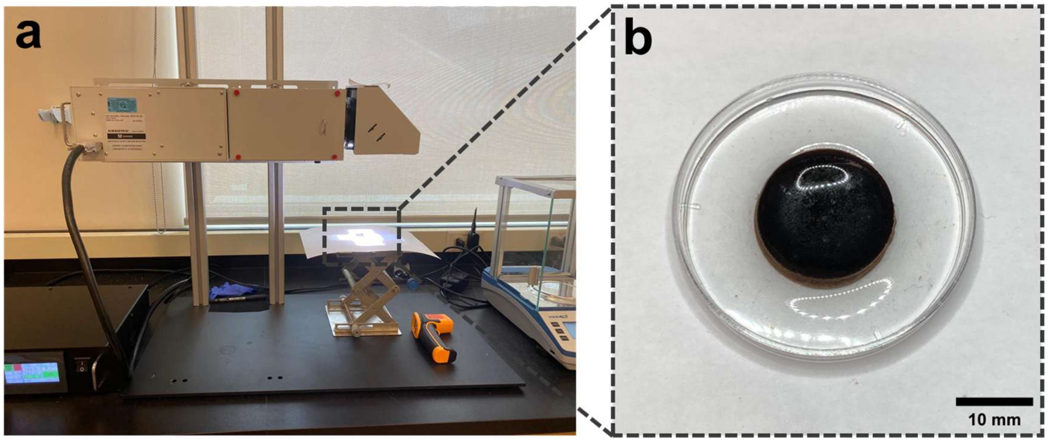

Figure S22. (a) Overview of the solar water evaporation testing system set-up. (b) Floating of the HTCAs in water.

S3. Supporting References

[1] Y. S. Vidya, B. N. Lakshminarasappa, *Phys. Res. Int.* **2013**, *1*, 641631.

[2] F. Gong, W. Wang, H. Li, D. Xia, Q. Dai, X. Wu, M. Wang, J. Li, D. V. Papavassiliou, R. Xiao, *Appl. Energy* **2020**, *261*, 114410.
